# Supplementary material for: Erb-b2 Receptor Tyrosine Kinase 2 (ERBB2) Promotes ATG12-Dependent Autophagy Contributing to Treatment Resistance of Breast Cancer Cells
Source: Cancers (Basel). 2021 Mar 2;13(5):1038. doi: 10.3390/cancers13051038 (PMC7958130; doi:10.3390/cancers13051038)
Supplement: Supplementary file 1 [file cancers-13-01038-s001.zip › supplementary material/cancers-1121649-final-suppl.docx]

Supplementary Material: Erb-b2 Receptor Tyrosine Kinase 2 (ERBB2) Promotes ATG12-Dependent Autophagy Contributing to Treatment Resistance of Breast Cancer Cells

Yongqiang Chen, Ruobing Wang, Shujun Huang, Elizabeth S. Henson, Jayce B and Spencer B. Gibson


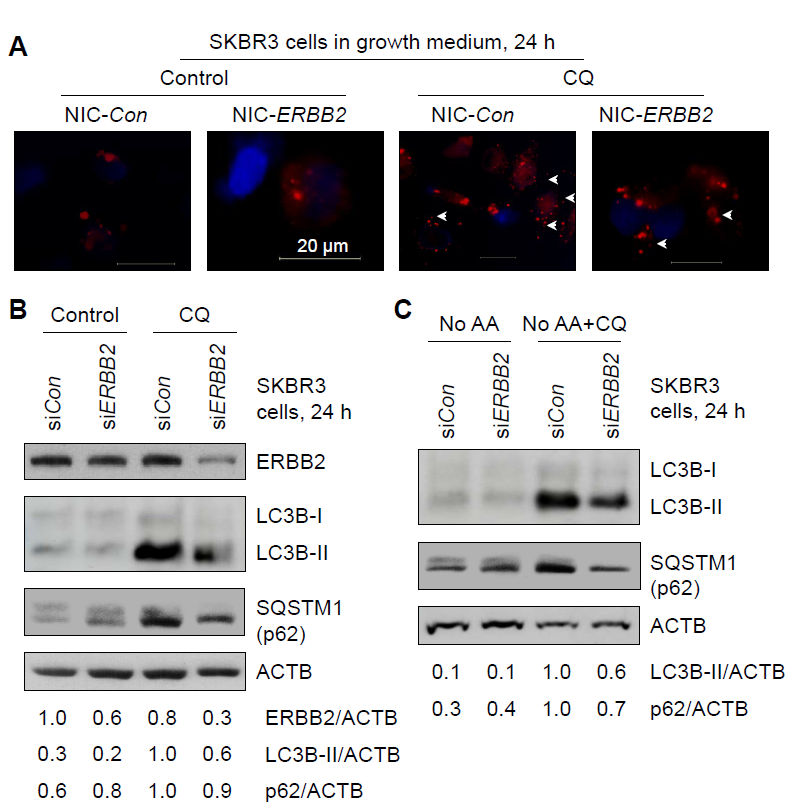


**Figure S1.** Knockout/knockdown of ERBB2 inhibits autophagyin SKBR3 cells**.** (**A**) More example images of mRFP-LC3 puncta(arrows) in SKBR3 cells without (NIC-*Con*) and with (NIC-*ERBB2*) ERBB2 knockout. (**B**) Basal autophagyinhibition by *ERBB2* siRNAknockdown. (**C**) Inhibition of amino acid starvation (No AA)-induced autophagyby *ERBB2*siRNAknockdown. Autophagywas measured by western blotting the autophagymarker protein LC3-II and the autophagysubstrate SQSTM1 (p62) in the absence and presence of chloroquine(CQ, 20 μM).Uncroppedwestern blot images and quantification of the protein band intensities were shown in File S1.


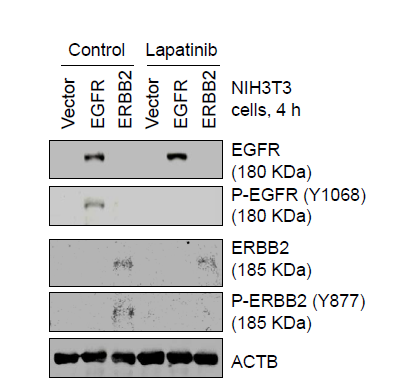


**Figure S2.** Western blot showed the expression of EGFR and ERBB2 and lapatinib (1 μM) inhibition of tyrosine (Y) phosphorylationof EGFR (at Y1068) and ERBB2 (Y877). Uncroppedwestern blot images were shown in File S1.


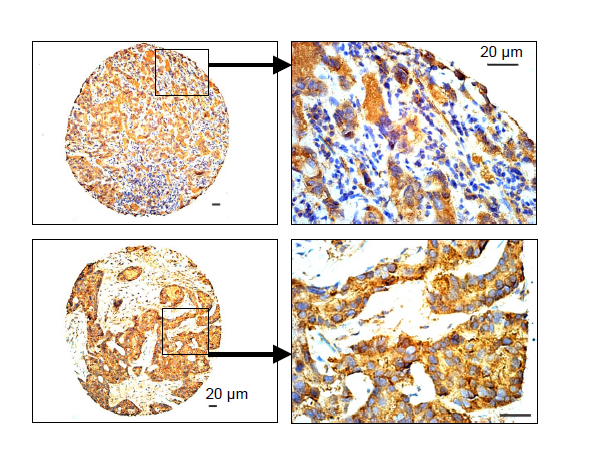


**Figure S3.** ATG12 protein is expressed in human breast cancer tissues. Immunohistochemistry (IHC) staining of ATG12 (brown) was performed using a tumor microarray slide prepared by the Manitoba Tumor Bank. These formalin-fixed, paraffin-embedded human breast tumour tissues were stained with an anti-ATG12 antibody(ab109491) and counter-stained with hematoxylin(blue) using the Ventana Autostainer following standard protocols provided by the manufacturer. These images are representative of the staining seen throughout the microarray.

**Figure S4.** Over-expression of ERBB2 increased *ATG12*mRNA expression. The mRNA level of *ATG12*was up-regulated by over-expression of ERBB2 in MDA-MB-231 cells. ERBB2 over-expression was demonstrated by western blot in Figure 2A. The ATG12 mRNA level was measured by real-time PCR. *, *p* < 0.05.


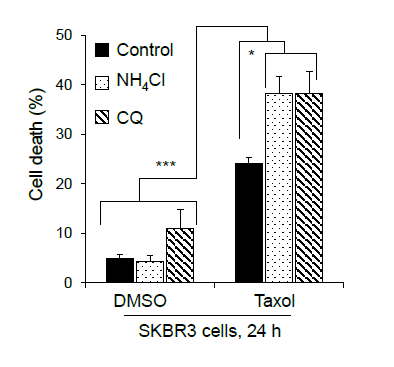


**Figure S5.** Autophagy inhibitors ammonium chloride (NH4Cl) and chloroquine(CQ) increased Taxol-induced cell death in SKBR3 breast cancer cells. Taxol (paclitaxel), 10 μM; NH4Cl, 30 mM; CQ, 20 μM. *, *p* < 0.05; ***, *p* < 0.001.


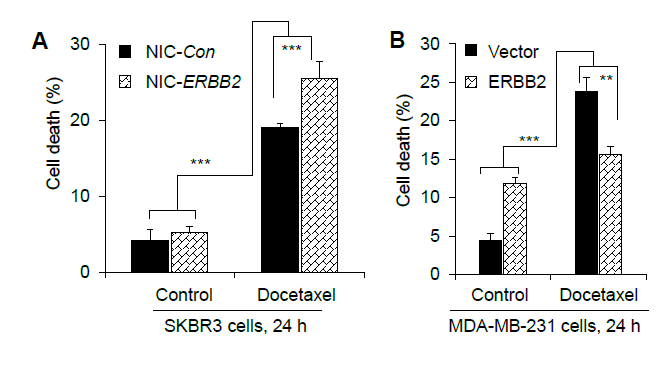


**Figure S6.** ERBB2 inhibited docetaxel-induced cell death in breast cancer cells. (**A**) ERBB2 knockout increased docetaxel-induced cell death in SKBR3 cells. ERBB2 knockout was demonstrated in Figure 1A. The “Control” data shared the “Control” data in Figure 7B. (**B**) ERBB2 over-expression decreased docetaxel-induced cell death in MDA-MB-231 cells. ERBB2 over-expression was demonstrated in Figure 2A. The “Control” data shared the “Control” data in Figure 7D. Docetaxel, 5 μM. ***, *p* < 0.001.


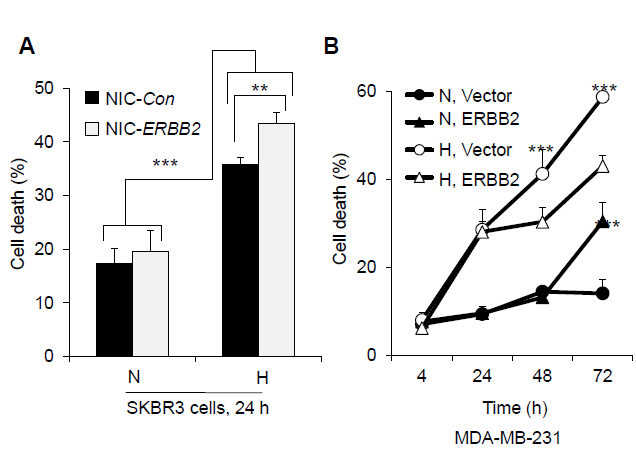


**Figure S7.** ERBB2 expression inhibited hypoxia-induced cell death. For hypoxia treatment, cells were maintained under hypoxic conditions (<1% oxygen) at 37°C within a hypoxic chamber (Forma Scientific, Waltham, MA) filled with 5% CO2balanced with N2. (**A**) ERBB2 knockout increased hypoxia-induced cell death in SKBR3 cells. Knockout of ERBB2 was demonstrated in Figure 1A. (**B**) ERBB2 over-expression decreased hypoxia-induced cell death in MDA-MB-231 cells. ERBB2 over-expression was demonstrated in Figure 2A. Results represent three independent 6-replicate experiments. *n*, normoxia; H, hypoxia. **p* < 0.05; ***p* < 0.01; ****p* < 0.001.

**Table S1.** Effects of autophagy gene upregulation on relapse-free survival (RFS)-based survival probability of patients with ERBB2-positive and –negative breast tumors.

| **Gene** | **Reporter** | **+** | **−** | **Gene** | **Reporter** | **+** | **−** |
| --- | --- | --- | --- | --- | --- | --- | --- |
| *ULK1* | 209333_at | NE | NE | *ATG4C** | 228190_at | NE | NE |
| *ULK2* | 204062_s_at | NE | NE | *ATG4D** | 226871_s_at | NE | NE |
| *FIP200* | 202033_s_at | NE | NE | *ATG5* | 202511_s_at | NE | Low |
| *ATG101* | 218214_at | NE | NE | *ATG7* | 218673_s_at | NE | NE |
| *ATG9A* | 202492_at | NE | Low | *ATG10* | 207774_at | NE | NE |
| *ATG9B** | 229252_at | NE | NE | *ATG12* | 213026_at | Low | NE |
| *BECN1* | 208946_s_at | NE | High | *ATG16L1* | 220521_s_at | NE | High |
| *PIK3CB* | 212688_at | NE | Low | *ATG16L2** | 225883_at | NE | High |
| *PIK3C3* | 204297_at | NE | NE | *MAP1LC3A** | 232011_s_at | High | High |
| *AMBRA1* | 52731_at | NE | NE | *MAP1LC3B* | 208786_s_at | Low | NE |
| *UVRAG* | 203241_at | NE | High | *MAP1LC3C* | 221697_at | High | NE |
| *BIF1* | 205340_at | NE | NE | *GABARAPL1* | 208868_s_at | NE | NE |
| *PIK3R4* | 212740_at | NE | NE | *GABARAPL2* | 209046_s_at | NE | NE |
| *ATG2A* | 213300_at | NE | NE | *WIPI1* | 203827_at | NE | NE |
| *ATG2B* | 219164_s_at | NE | NE | *ATG18B* | 202031_s_at | NE | NE |
| *ATG3* | 221492_s_at | NE | Low | *VMP1* | 220990_s_at | NE | NE |
| *ATG4A* | 213115_at | NE | NE | *SQSTM1* | 201471_s_at | NE | NE |
| *ATG4B* | 204902_s_at | NE | NE |  |  |  |  |

Data were extracted from the Kaplan-Meier Plotter breast cancer datasets (http://kmplot.com/analysis/index.php?p = ser-vice&cancer = breast, accessed on 19 November 2019). Number of patients (*n*): *n* = 252 (* *n* = 150) with ERBB2-positive ( + ) breast tumors, *n* = 800 (* *n* = 635) with ERBB2-negative (−) breast tumors. Statistical significance between survival probabilities of patients bearing breast tumors with high-and low-levels of an autophagy gene: NE, no significant difference; High, sig-nificantly higher survival probability with high expression of the gene; Low, significantly lower survival probability with high expression of the gene. Examples of the plots related to ATG12, MAP1LC3B and BECN1were demonstrated in Figure 5. The plots of other autophagy genes were not shown. Gene names were italicized to distinguish them from protein names.
