# Supplementary material for: Erb-b2 Receptor Tyrosine Kinase 2 (ERBB2) Promotes ATG12-Dependent Autophagy Contributing to Treatment Resistance of Breast Cancer Cells
Source: Cancers (Basel). 2021 Mar 2;13(5):1038. doi: 10.3390/cancers13051038 (PMC7958130; doi:10.3390/cancers13051038)
Supplement: Supplementary file 1 [file cancers-13-01038-s001.zip › supplementary material/File S1. Uncropped Western blots.pdf]

Uncropped Western blots for Figure S1B

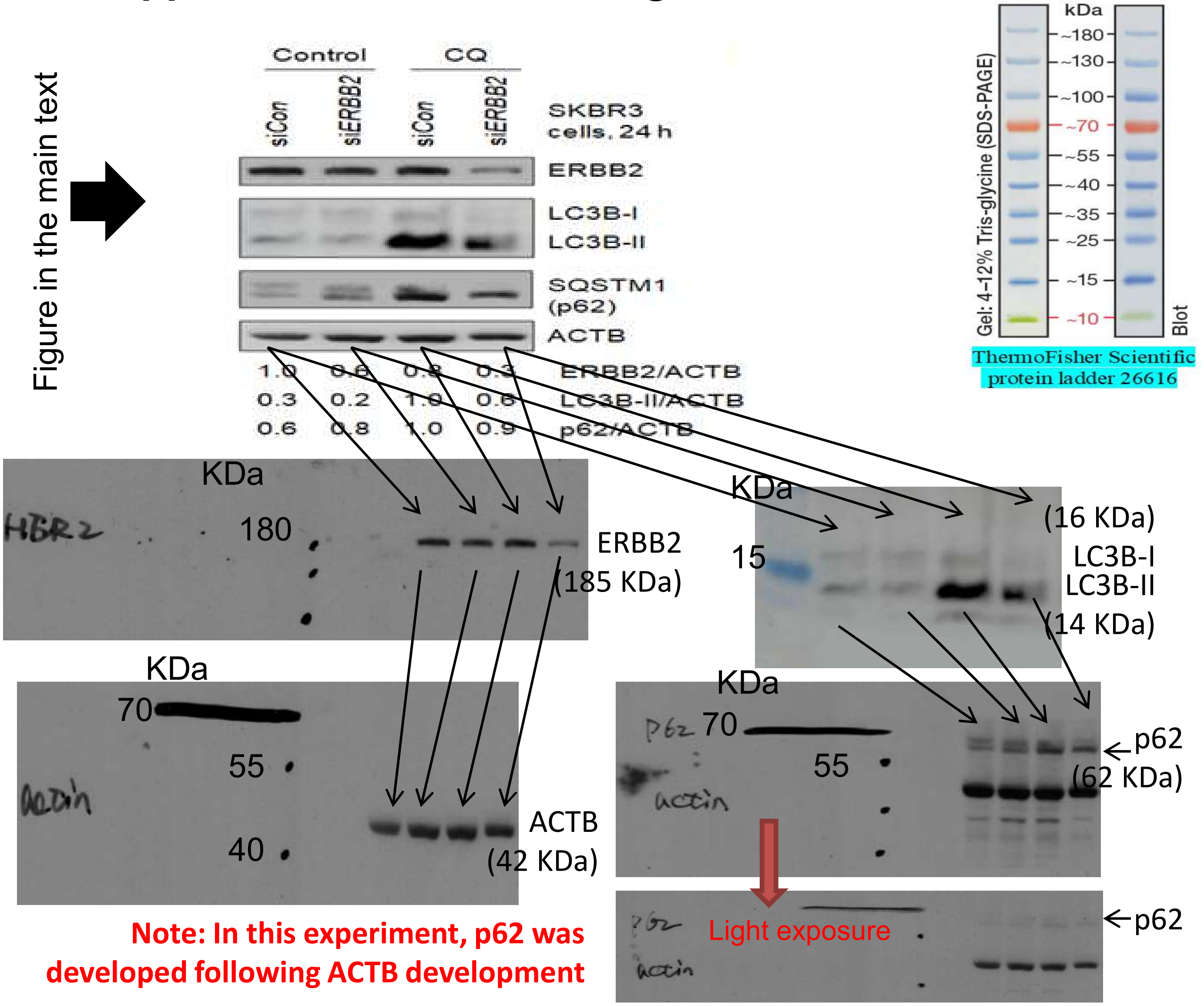

24 h	Area	ACTB Intensity	ACTB Intensity	Area	ERBB2 Intensity	ERBB2 Intensity	ERBB2/ACTB	Relative
SKBR3		Mean	(-background)		Mean	(-background)		
background	9424	124.097		6448	121.333			
siCon	9424	136.293	12.196	6448	155.347	34.014	2.7889472	1
siERBB2	9424	140.668	16.571	6448	150.044	28.711	1.73260515	0.6212399
siCon+CQ	9424	140.435	16.338	6448	156.412	35.079	2.14708043	0.7698534
siERBB2+CQ	9424	137.596	13.499	6448	134.44	13.107	0.97096081	0.348146
24 h	Area	ACTB Intensity	ACTB Intensity	Area	LC3II Intensity	LC3II Intensity	LC3II/ACTB	Relative
SKBR3		Mean	(-background)		Mean	(-background)		
background	9424	124.097		3216	95.976			
siCon	9424	136.293	12.196	3216	111.514	15.538	1.27402427	0.2736692
siERBB2	9424	140.668	16.571	3216	110.434	14.458	0.87248808	0.1874165
siCon+CQ	9424	140.435	16.338	3216	172.035	76.059	4.65534337	1
siERBB2+CQ	9424	137.596	13.499	3216	136.478	40.502	3.0003704	0.6445003
24 h	Area	ACTB Intensity	ACTB Intensity	Area	SQSTM1(p62) Intensity	p62 Intensity	p62I/ACTB	Relative
SKBR3		Mean	(-background)		Mean	(-background)		
background	9424	124.097		5264	122.021			
siCon	9424	136.293	12.196	5264	152.474	30.453	2.49696622	0.6331156
siERBB2	9424	140.668	16.571	5264	166.413	44.392	2.67889687	0.6792448
siCon+CQ	9424	140.435	16.338	5264	186.457	64.436	3.94393439	1
siERBB2+CQ	9424	137.596	13.499	5264	168.886	46.865	3.47173865	0.8802729

Uncropped Western blots for Figure S1C

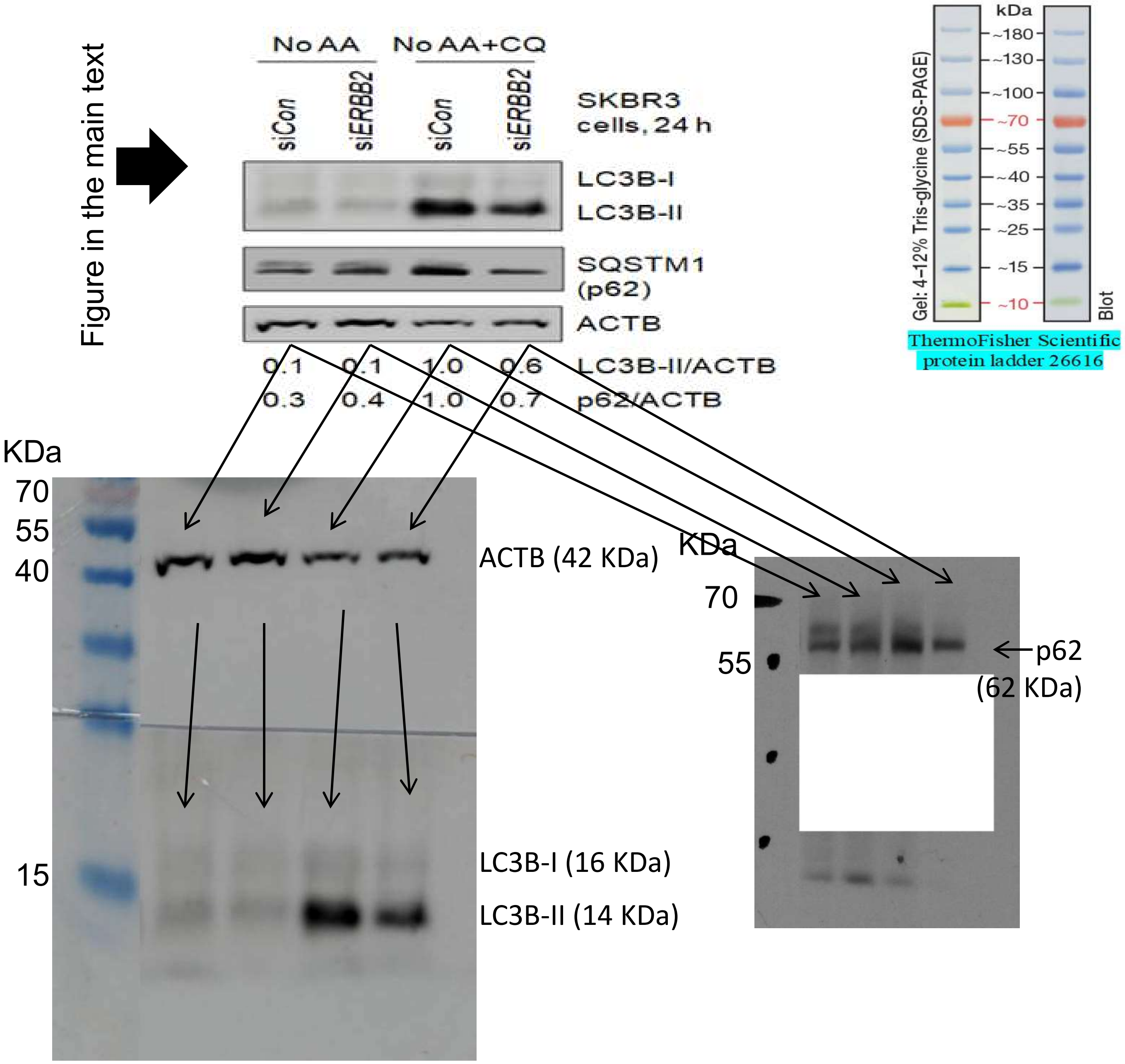

24 h	Area	ACTB Intensity	ACTB Intensity	Area	LC3II Intensity	LC3II Intensity	LC3II/ACTB	Relative
SKBR3		Mean	(-background)		Mean	(-background)		
background	9424	124.097		3216	95.976			
siCon+No AA	9424	145.568	21.471	3216	113.772	17.796	0.8288389	0.1094923
siERBB2+No AA	9424	142.014	17.917	3216	112.623	16.647	0.9291176	0.1227395
siCon+No AA+CQ	9424	132.703	8.606	3216	161.122	65.146	7.569835	1
siERBB2+No AA+CQ	9424	132.414	8.317	3216	136.273	40.297	4.84513647	0.6400584
		ACTB	ACTB		SQSTM1(p62)	p62	p62I/ACTB	
24 h	Area	Intensity	Intensity	Area	Intensity	Intensity		Relative
SKBR3		Mean	(-background)		Mean	(-background)		
background	9424	124.097		5264	122.021			
siCon+No AA	9424	145.568	21.471	5264	170.659	48.638	2.26528806	0.2589537
siERBB2+No AA	9424	142.014	17.917	5264	181.625	59.604	3.32667299	0.3802846
siCon+No AA+CQ	9424	132.703	8.606	5264	197.305	75.284	8.74785034	1
siERBB2+No AA+CQ	9424	132.414	8.317	5264	170.373	48.352	5.81363472	0.6645787

Uncropped Western blots for Figure S2

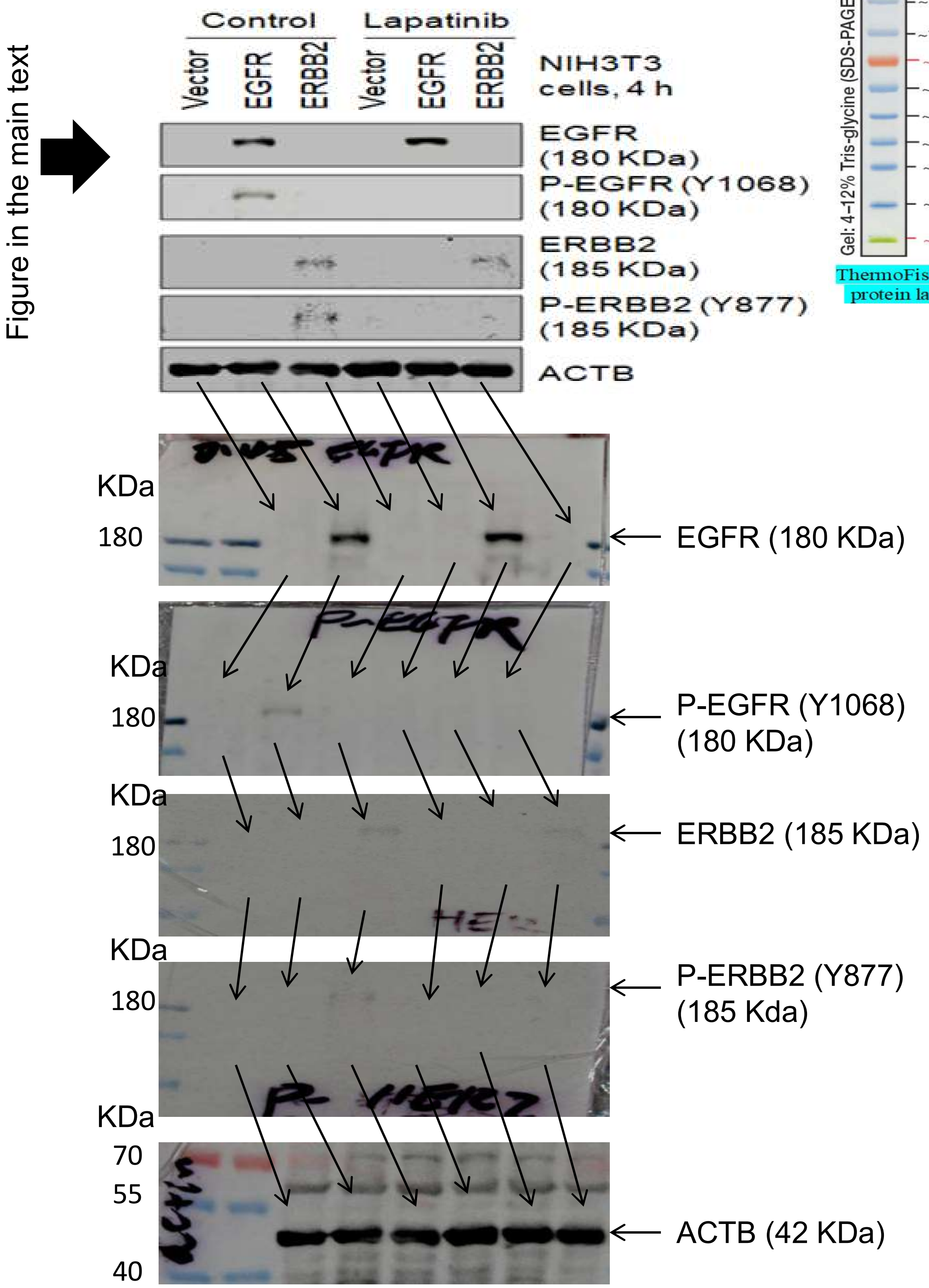

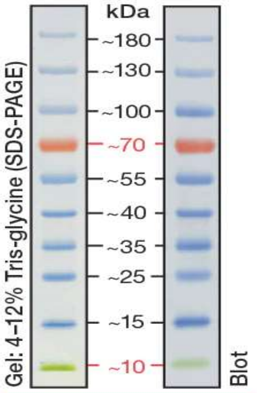

ThermoFisher Scientific protein ladder 26616

Uncropped Western blots for Figure 1A

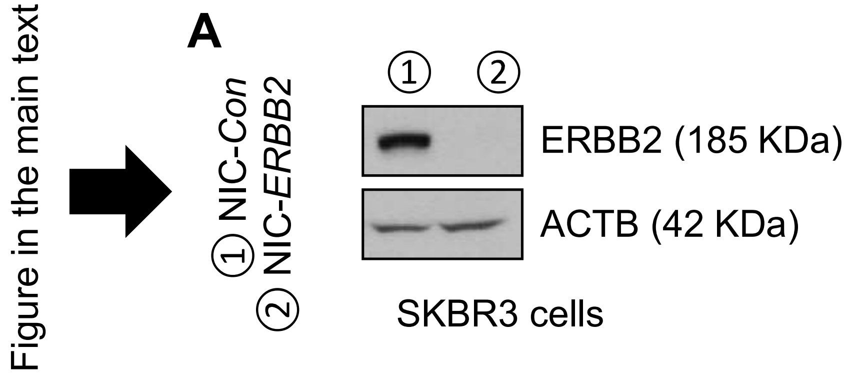

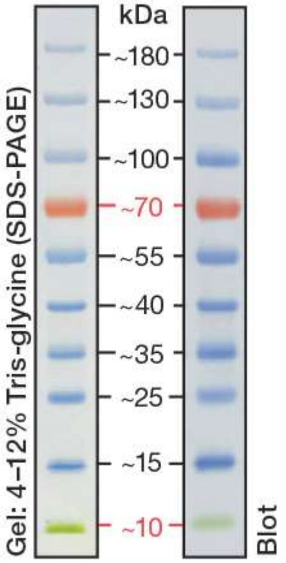

ThermoFisher Scientific protein ladder 26616

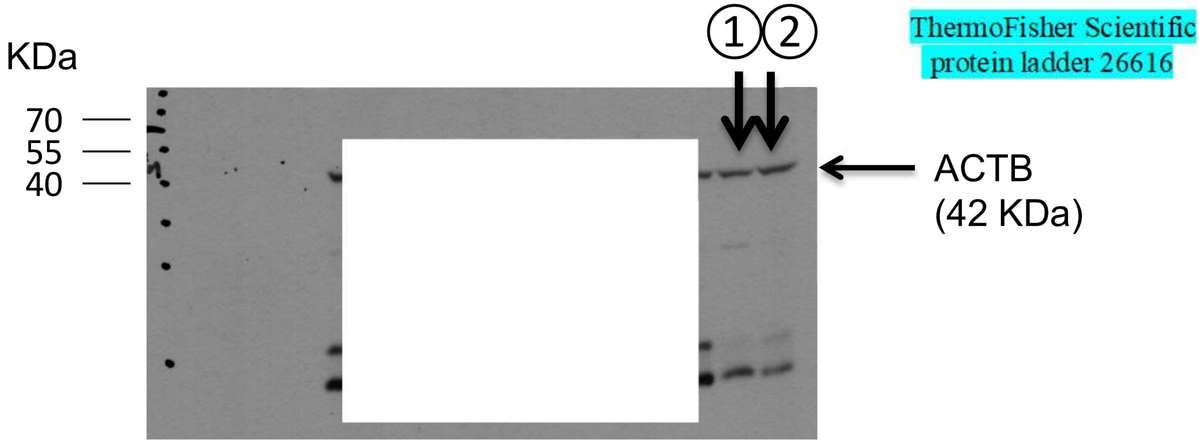

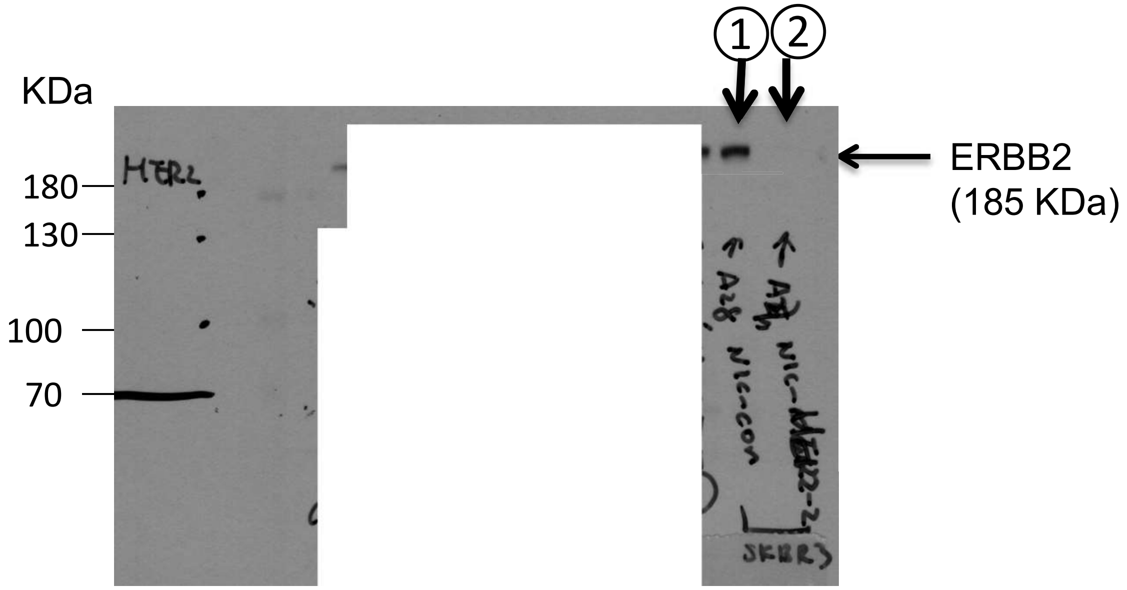

Uncropped Western blots for Figure 1A bar graph-data set 1

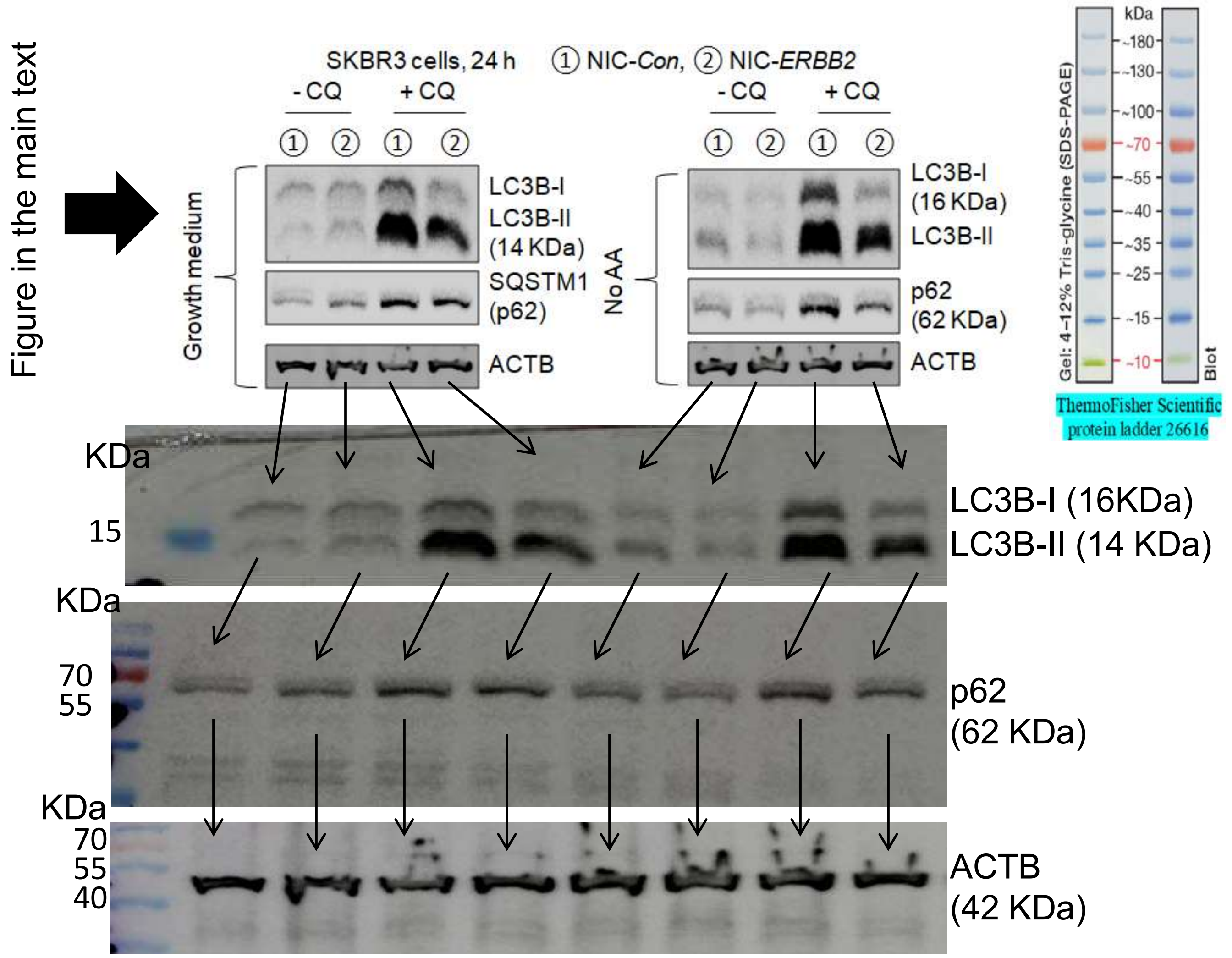

Set 1			ACTB	ACTB		LC3II	LC3II	LC3II/ACTB	
			Intensity	Intensity		Intensity	Intensity		Relative
		Area	Mean	(-background)	Area	Mean	(-background)		
SKBR3	Background	2520	92.427		2072	129			
Control	NIC-Con	2520	147.011	54.584	2072	135.114	6.114	0.11201085	0.068371
Control	NIC-ERBB2	2520	156.341	63.914	2072	144.008	15.008	0.23481553	0.14333
CQ	NIC-Con	2520	140.901	48.474	2072	208.414	79.414	1.63828032	1
CQ	NIC-ERBB2	2520	155.352	62.925	2072	183.202	54.202	0.86137465	0.52578
			ACTB	ACTB		p62	p62	p62/ACTB	
			Intensity	Intensity		Intensity	Intensity		Relative
		Area	Mean	(-background)	Area	Mean	(-background)		
SKBR3	Background	2520	92.427		1624	127.868			
Control	NIC-Con	2520	147.011	54.584	1624	149.19	21.322	0.39062729	0.635454
Control	NIC-ERBB2	2520	156.341	63.914	1624	150.285	22.417	0.35073693	0.570563
CQ	NIC-Con	2520	140.901	48.474	1624	157.666	29.798	0.61472129	1
CQ	NIC-ERBB2	2520	155.352	62.925	1624	153.975	26.107	0.41489074	0.674925
			ACTB	ACTB		LC3II	LC3II	LC3II/ACTB	
			Intensity	Intensity		Intensity	Intensity		Relative
		Area	Mean	(-background)	Area	Mean	(-background)		
SKBR3	Background	2520	92.427		2072	129			
No AA	NIC-Con	2520	160.589	68.162	2072	145.121	16.121	0.23651008	0.195531
No AA	NIC-ERBB2	2520	158.954	66.527	2072	140.78	11.78	0.17707096	0.14639
No AA+CQ	NIC-Con	2520	153.95	61.523	2072	203.417	74.417	1.20958016	1
No AA+CQ	NIC-ERBB2	2520	149.183	56.756	2072	172.809	43.809	0.77188315	0.638141
			ACTB	ACTB		p62	p62	p62/ACTB	
			Intensity	Intensity		Intensity	Intensity		Relative
		Area	Mean	(-background)	Area	Mean	(-background)		
SKBR3	Background	2520	92.427		1624	127.868			
No AA	NIC-Con	2520	160.589	68.162	1624	147.222	19.354	0.2839412	0.576475
No AA	NIC-ERBB2	2520	158.954	66.527	1624	144.181	16.313	0.24520871	0.497838
No AA+CQ	NIC-Con	2520	153.95	61.523	1624	158.171	30.303	0.4925475	1
No AA+CQ	NIC-ERBB2	2520	149.183	56.756	1624	148.515	20.647	0.36378533	0.738579

Uncropped Western blots for Figure 1A bar graph-data set 2

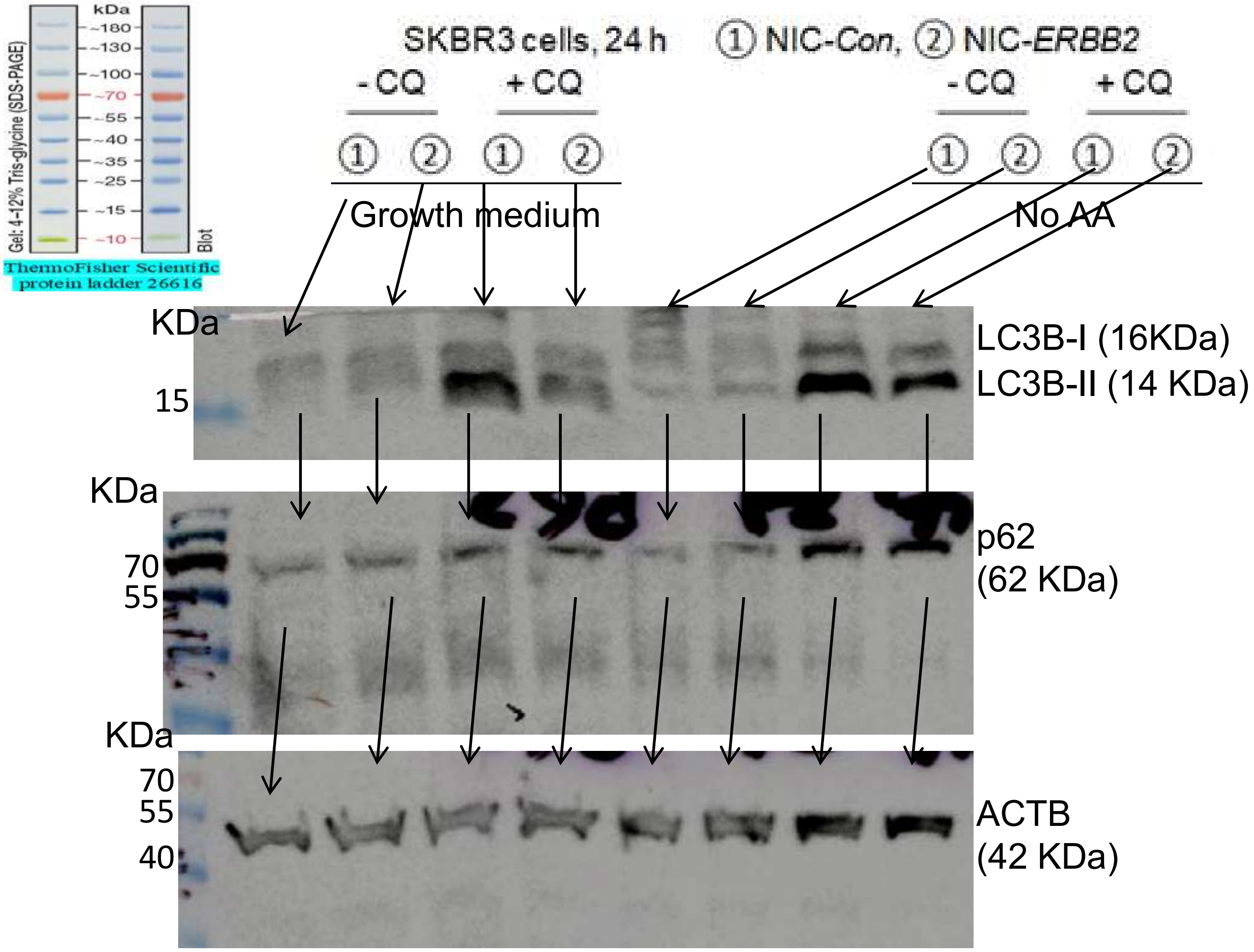

Set 2			ACTB	ACTB		LC3II	LC3II	LC3II/ACTB	
			Intensity	Intensity		Intensity	Intensity		Relative
		Area	Mean	(-background)	Area	Mean	(-background)		
SKBR3	Background	1856	100.922		2940	98.075			
Control	NIC-Con	1856	132.922	32	2940	106.207	8.132	0.254125	0.135395
Control	NIC-ERBB2	1856	130.711	29.789	2940	109.973	11.898	0.39940918	0.2128
CQ	NIC-Con	1856	126.085	25.163	2940	145.304	47.229	1.87692247	1
CQ	NIC-ERBB2	1856	133.341	32.419	2940	120.869	22.794	0.7031062	0.374606
			ACTB	ACTB		p62	p62	p62/ACTB	
			Intensity	Intensity		Intensity	Intensity		Relative
		Area	Mean	(-background)	Area	Mean	(-background)		
SKBR3	Background	1856	100.922		3266	97.994			
Control	NIC-Con	1856	132.922	32	3266	105.155	7.161	0.22378125	0.074508
Control	NIC-ERBB2	1856	130.711	29.789	3266	110.055	12.061	0.404881	0.134805
CQ	NIC-Con	1856	126.085	25.163	3266	173.57	75.576	3.00345746	1
CQ	NIC-ERBB2	1856	133.341	32.419	3266	131.697	33.703	1.0396064	0.346137
			ACTB	ACTB		LC3II	LC3II	LC3II/ACTB	
			Intensity	Intensity		Intensity	Intensity		Relative
		Area	Mean	(-background)	Area	Mean	(-background)		
SKBR3	Background	1856	100.922		2940	98.075			
No AA	NIC-Con	1856	129.539	28.617	2940	101.484	3.409	0.119125	0.075218
No AA	NIC-ERBB2	1856	137.82	36.898	2940	103.588	5.513	0.14941189	0.094342
No AA+CQ	NIC-Con	1856	147.701	46.779	2940	172.16	74.085	1.58372347	1
No AA+CQ	NIC-ERBB2	1856	145.685	44.763	2940	134.051	35.976	0.80369948	0.507475
			ACTB	ACTB		p62	p62	p62/ACTB	
			Intensity	Intensity		Intensity	Intensity		Relative
		Area	Mean	(-background)	Area	Mean	(-background)		
SKBR3	Background	1856	100.922		3266	97.994			
No AA	NIC-Con	1856	129.539	28.617	3266	105.173	7.179	0.25086487	0.180214
No AA	NIC-ERBB2	1856	137.82	36.898	3266	108.398	10.404	0.2819665	0.202557
No AA+CQ	NIC-Con	1856	147.701	46.779	3266	163.112	65.118	1.39203489	1
No AA+CQ	NIC-ERBB2	1856	145.685	44.763	3266	139.703	41.709	0.93177401	0.669361

Uncropped Western blots for Figure 1A bar graph-data set 3

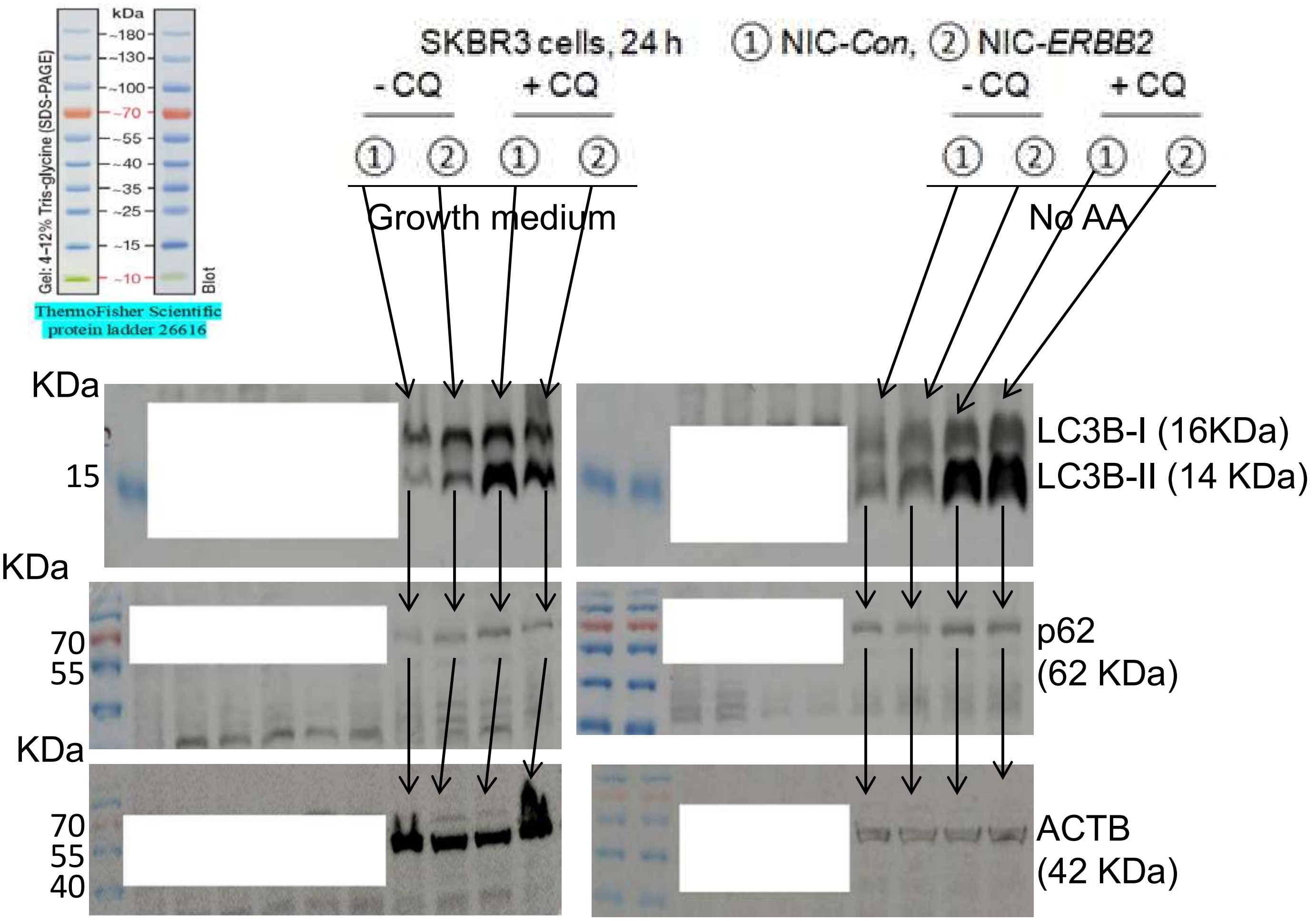

Set 3			ACTB	ACTB		LC3II	LC3II	LC3II/ACTB	
			Intensity	Intensity		Intensity	Intensity		Relative
		Area	Mean	(-background)	Area	Mean	(-background)		
SKBR3	Background	1800	111.676		2562	94.411			
Control	NIC-Con	1800	191.449	79.773	2562	106.689	12.278	0.15391172	0.111294
Control	NIC-ERBB2	1800	179.309	67.633	2562	123.616	29.205	0.43181583	0.312248
CQ	NIC-Con	1800	173.215	61.539	2562	179.515	85.104	1.3829279	1
CQ	NIC-ERBB2	1800	203.258	91.582	2562	159.734	65.323	0.71327335	0.51577
			ACTB	ACTB		p62	p62	p62/ACTB	
			Intensity	Intensity		Intensity	Intensity		Relative
		Area	Mean	(-background)	Area	Mean	(-background)		
SKBR3	Background	1800	111.676		816	94.596			
Control	NIC-Con	1800	191.449	79.773	816	106.96	12.364	0.15498978	0.305653
Control	NIC-ERBB2	1800	179.309	67.633	816	115.588	20.992	0.31038103	0.612099
CQ	NIC-Con	1800	173.215	61.539	816	125.801	31.205	0.50707681	1
CQ	NIC-ERBB2	1800	203.258	91.582	816	114.676	20.08	0.21925706	0.432394
			ACTB	ACTB		LC3II	LC3II	LC3II/ACTB	
			Intensity	Intensity		Intensity	Intensity		Relative
		Area	Mean	(-background)	Area	Mean	(-background)		
SKBR3	Background	2288	111.493		3780	96.298			
No AA	NIC-Con	2288	121.625	10.132	3780	116.858	20.56	2.02921437	0.238002
No AA	NIC-ERBB2	2288	119.541	8.048	3780	125.336	29.038	3.60810139	0.423185
No AA+CQ	NIC-Con	2288	120.819	9.326	3780	175.812	79.514	8.52605619	1
No AA+CQ	NIC-ERBB2	2288	126.024	14.531	3780	182.531	86.233	5.93441608	0.696033
			ACTB	ACTB		p62	p62	p62/ACTB	
			Intensity	Intensity		Intensity	Intensity		Relative
		Area	Mean	(-background)	Area	Mean	(-background)		
SKBR3	Background	2288	111.493		731	97.453			
No AA	NIC-Con	2288	121.625	10.132	731	119.476	22.023	2.17360837	0.544804
No AA	NIC-ERBB2	2288	119.541	8.048	731	115.893	18.44	2.29125249	0.574291
No AA+CQ	NIC-Con	2288	120.819	9.326	731	134.661	37.208	3.9897062	1
No AA+CQ	NIC-ERBB2	2288	126.024	14.531	731	129.402	31.949	2.19867869	0.551088

Uncropped Western blots for Figure 1A bar graph-data set 4

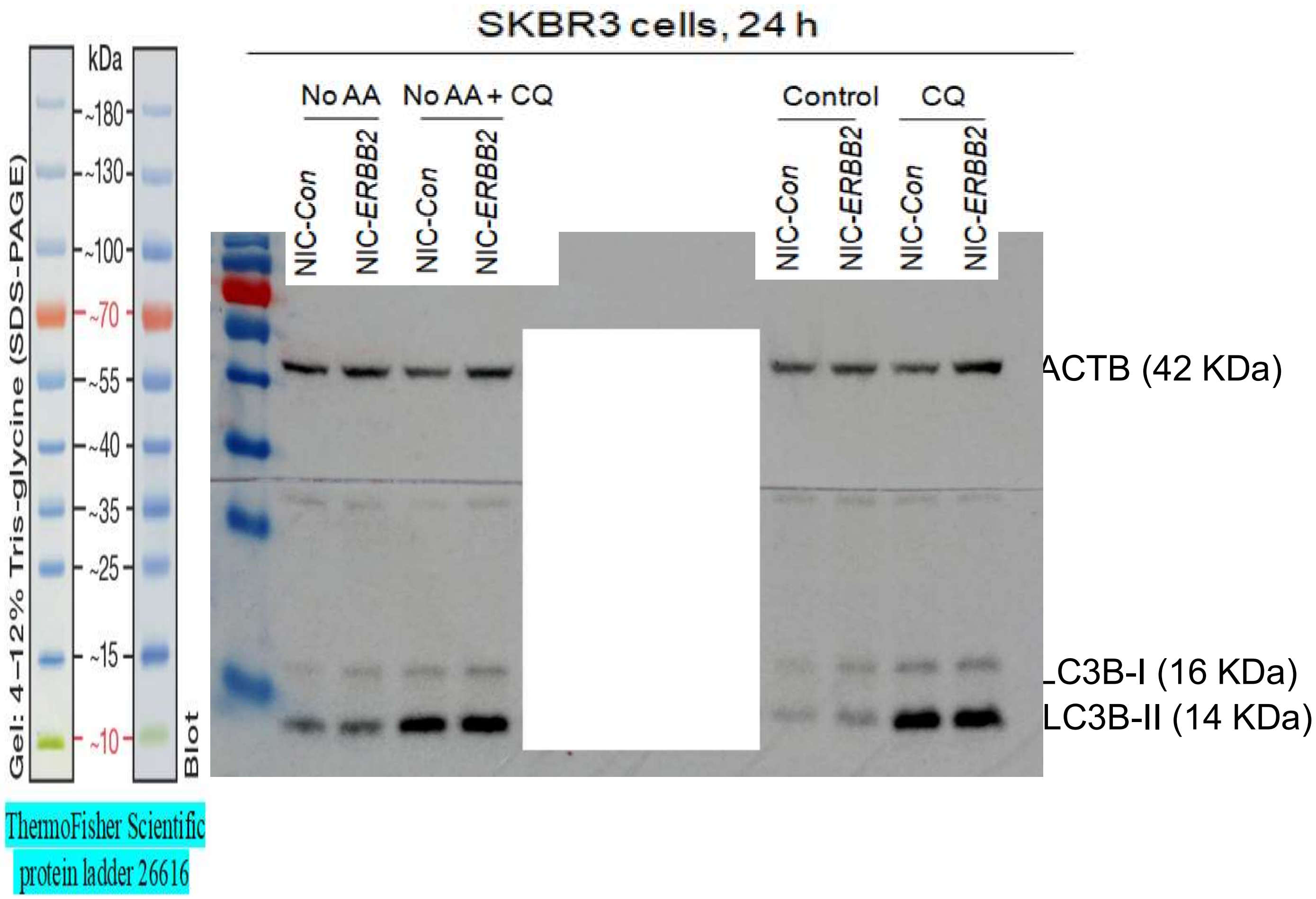

Set 4			ACTB	ACTB		LC3II	LC3II	LC3II/ACTB	
			Intensity	Intensity		Intensity	Intensity		Relative
		Area	Mean	(-background)	Area	Mean	(-background)		
SKBR3	Background	1288	91.355		1624	93.18			
Control	NIC-Con	1288	128.43	37.075	1624	102.255	9.075	0.24477411	0.096658
Control	NIC-ERBB2	1288	130.464	39.109	1624	108.432	15.252	0.38998696	0.154001
CQ	NIC-Con	1288	122.364	31.009	1624	171.706	78.526	2.53236157	1
CQ	NIC-ERBB2	1288	151.491	60.136	1624	172.465	79.285	1.31842823	0.520632
No AA	NIC-Con	1288	141.188	49.833	1624	122.922	29.742	0.59683342	0.272439
No AA	NIC-ERBB2	1288	143.619	52.264	1624	127.023	33.843	0.64753942	0.295585
No AA+CQ	NIC-Con	1288	123.578	32.223	1624	163.771	70.591	2.19070229	1
No AA+CQ	NIC-ERBB2	1288	139.143	47.788	1624	170.046	76.866	1.60847912	0.73423

Uncropped Western blots for Figure 1A—data set 5 for bar graph

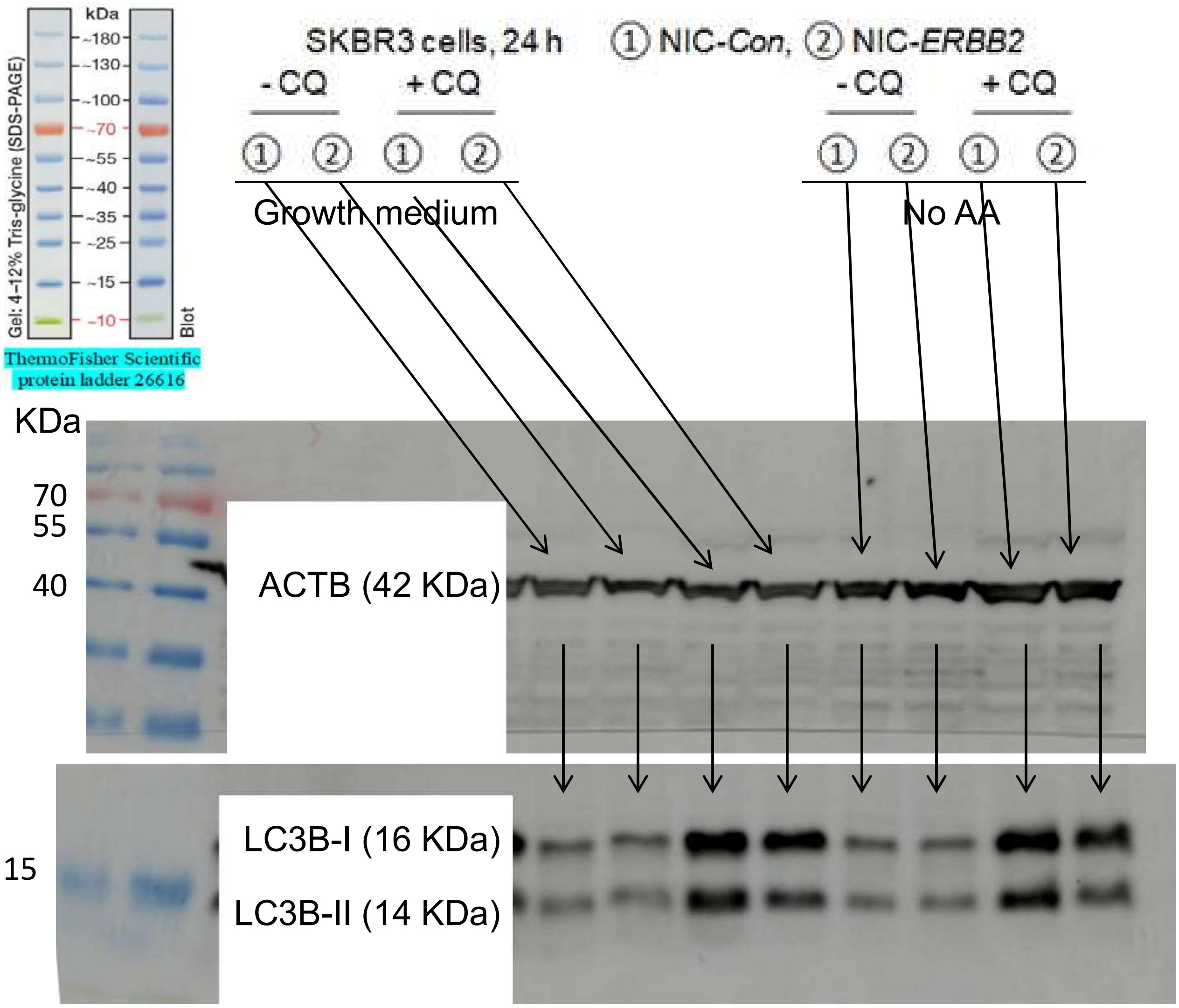

Set 5			ACTB Intensity Mean	ACTB Intensity (-background)		LC3II Intensity Mean	LC3II Intensity (-background)	LC3II/ACTB	Relative
		Area			Area				
SKBR3	Background	2048	93.457		2432	94.582			
Control	NIC-Con	2048	144.187	50.73	2432	122.211	27.629	0.54462842	0.516813
Control	NIC-ERBB2	2048	142.92	49.463	2432	122.316	27.734	0.56070194	0.532065
CQ	NIC-Con	2048	148.491	55.034	2432	152.578	57.996	1.05382127	1
CQ	NIC-ERBB2	2048	143.132	49.675	2432	132.431	37.849	0.76193256	0.723019
No AA	NIC-Con	2048	152.661	59.204	2432	114.011	19.429	0.32817039	0.457537
No AA	NIC-ERBB2	2048	159.542	66.085	2432	117.925	23.343	0.3532269	0.492471
No AA+CQ	NIC-Con	2048	167.849	74.392	2432	147.94	53.358	0.71725454	1
No AA+CQ	NIC-ERBB2	2048	161.312	67.855	2432	130.232	35.65	0.52538501	0.732495

Uncropped Western blots for Figure 1A for bar graph

SKBR3 24h	LC3II	Set(1)	Set(2)	Set(3)	Set(4)	Set(5)	mean	SD	p (vs NIC-con)	p (vs Control)
NIC-Con	Control	0.068371	0.1354	0.111294	0.096658	0.516813	0.1857062	0.186679697		
NIC-ERBB2	Control	0.14333	0.2128	0.312248	0.154001	0.532065	0.2708888	0.160648611		
NIC-Con	CQ	1	1	1	1	1	1	0		0.000618925
NIC-ERBB2	CQ	0.52578	0.37461	0.51577	0.520632	0.723019	0.5319614	0.124189829	0.001085728	0.022051437
	**p62**	**Set(1)**	**Set(2)**	**Set(3)**			mean	SD		
NIC-Con	Control	0.635454	0.07451	0.305653			0.338538333	0.281915213		
NIC-ERBB2	Control	0.570563	0.13481	0.612099			0.439155667	0.264392334		
NIC-Con	CQ	1	1	1			1	0		0.055551586
NIC-ERBB2	CQ	0.674925	0.34614	0.432394			0.484485333	0.170471457	0.034571107	0.817178121
SKBR3 24h	**LC3II**	**Set(1)**	**Set(2)**	**Set(3)**	**Set(4)**	**Set(5)**	mean	SD	p (vs NIC-con)	p (vs Control)
NIC-Con	No AA	0.195531	0.07522	0.238002	0.272439	0.457537	0.2477454	0.138932781		
NIC-ERBB2	No AA	0.14639	0.09434	0.423185	0.295585	0.492471	0.2903946	0.171514612		
NIC-Con	No AA+CQ	1	1	1	1	1	1	0		0.000266976
NIC-ERBB2	No AA+CQ	0.638141	0.50748	0.696033	0.73423	0.732495	0.6616748	0.094593422	0.001325423	0.005010992
SKBR3 24h	**p62**	**Set(1)**	**Set(2)**	**Set(3)**			mean	SD		
NIC-Con	No AA	0.576475	0.18021	0.544804			0.433831	0.220208878		
NIC-ERBB2	No AA	0.497838	0.20256	0.574291			0.424895333	0.196308472		
NIC-Con	No AA+CQ	1	1	1			1	0		0.046906618
NIC-ERBB2	No AA+CQ	0.738579	0.66936	0.551088			0.653009333	0.094809027	0.023993286	0.171249962

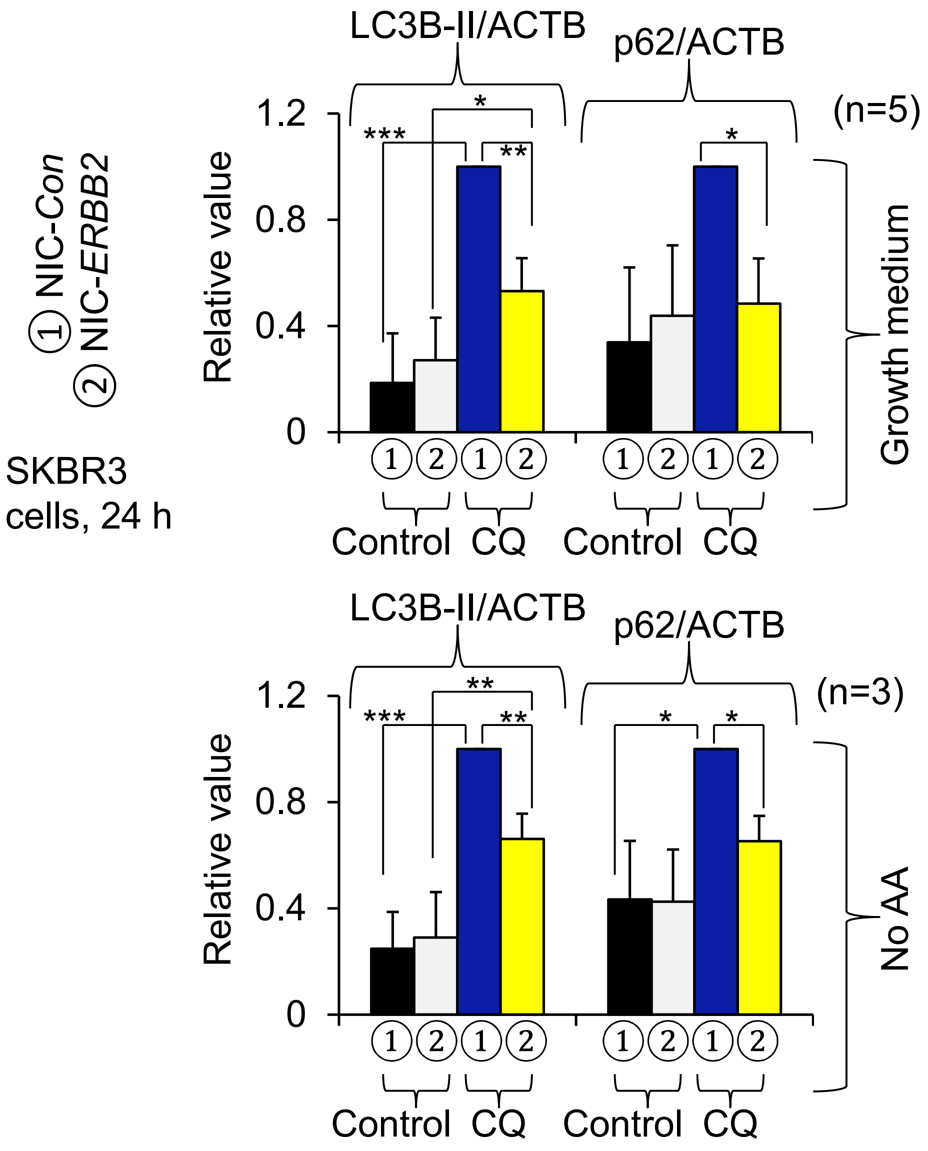

Uncropped Western blots for Figure 2A

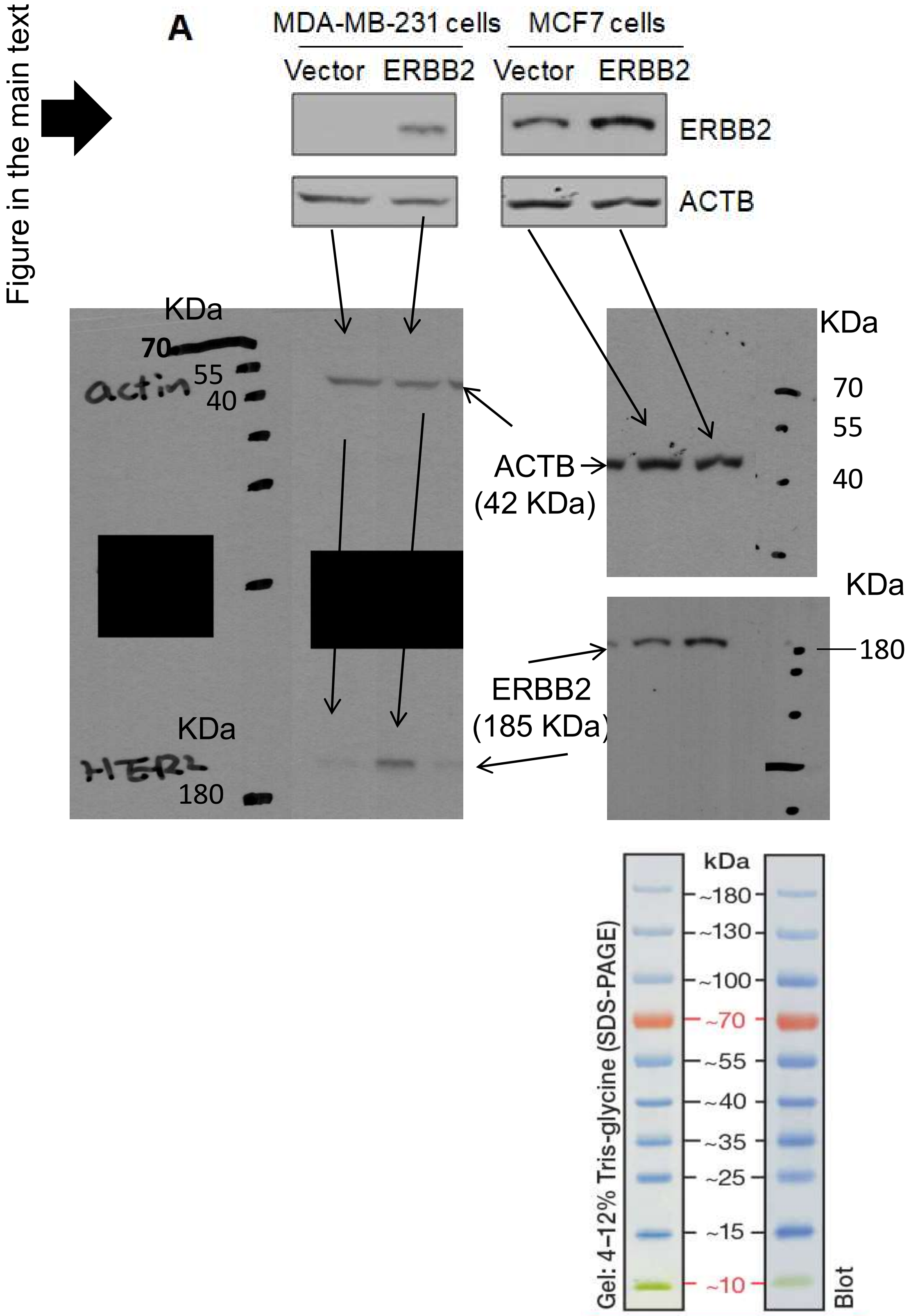

ThermoFisher Scientific
protein ladder 26616

Uncropped Western blots for Figure 2B

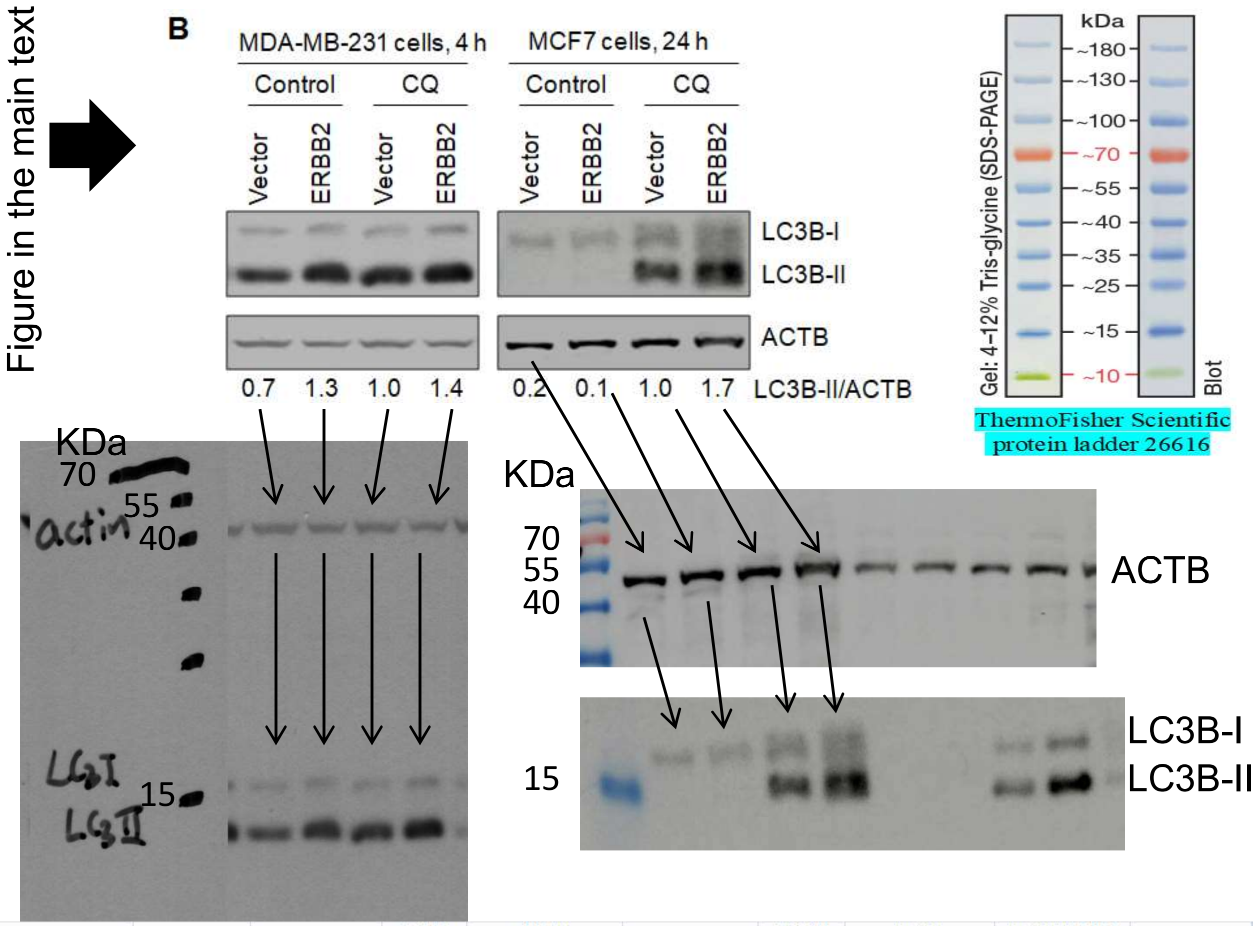

			ACTB	ACTB		LC3II	LC3II	LC3II/ACTB	
			Intensity	Intensity		Intensity	Intensity		Relative
MDA-MB-231 cells		Area	Mean	(-background)	Area	Mean	(-background)		
4 h	background	6720	115.056		7616	114.595			
Vector	Control	6720	136.853	21.797	7616	144.58	29.985	1.375648025	0.67704661
HER2	Control	6720	130.841	15.785	7616	157.63	43.035	2.726322458	1.34180208
Vector	CQ(20uM)	6720	136.635	21.579	7616	158.44	43.845	2.031836508	1
HER2	CQ	6720	133.459	18.403	7616	168.402	53.807	2.923816769	1.43900199
			ACTB	ACTB		LC3II	LC3II	LC3II/ACTB	
			Intensity	Intensity		Intensity	Intensity		Relative
MCF7 cells		Area	Mean	(-background)	Area	Mean	(-background)		
24 h	background	1525	86.711		3074	95.655			
Vector	Control	1525	140.095	53.384	3074	100.424	4.769	0.089333883	0.16769361
HER2	Control	1525	147.833	61.122	3074	99.015	3.36	0.054972023	0.10319105
Vector	CQ(20uM)	1525	153.87	67.159	3074	131.432	35.777	0.532720856	1
HER2	CQ	1525	155.728	69.017	3074	158.001	62.346	0.903342655	1.69571483

Uncropped Western blots for Figure 2C

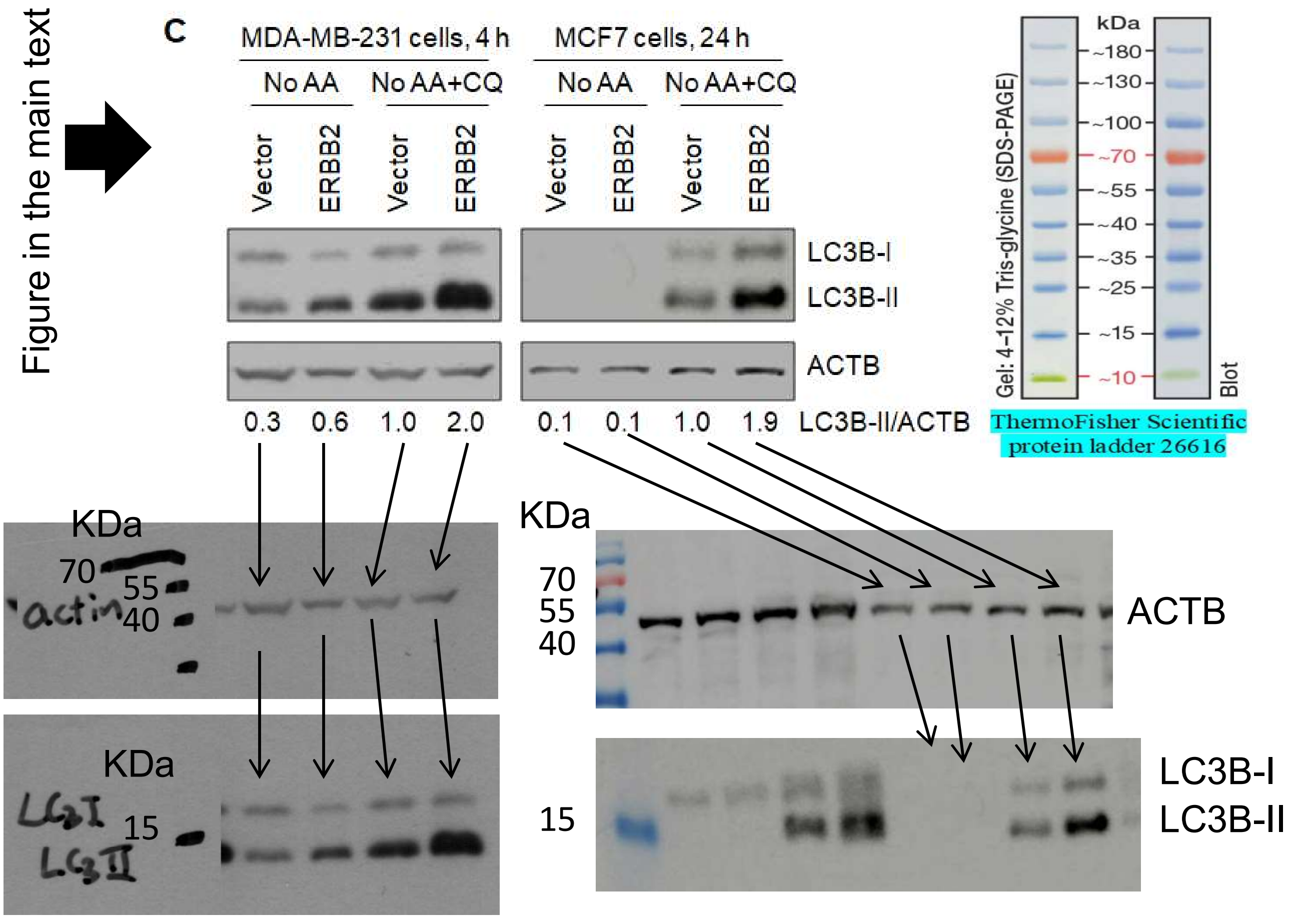

			ACTB	ACTB		LC3II	LC3II	LC3II/ACTB	
			Intensity	Intensity		Intensity	Intensity		Relative
MDA-MB-231 CELLs		Area	Mean	(-background)	Area	Mean	(-background)		
4 h	background	6720	115.056		7616	114.595			
Vector	No AA	6720	145.961	30.905	7616	125.742	11.147	0.360685973	0.32832791
HER2	No AA	6720	138.08	23.024	7616	128.842	14.247	0.61878909	0.56327593
Vector	No AA+CQ	6720	139.885	24.829	7616	141.871	27.276	1.09855411	1
HER2	No AA+CQ	6720	139.799	24.743	7616	168.485	53.89	2.177989734	1.98259668
			ACTB	ACTB		LC3II	LC3II	LC3II/ACTB	
			Intensity	Intensity		Intensity	Intensity		Relative
MCF7 cells		Area	Mean	(-background)	Area	Mean	(-background)		
24 h	background	1525	86.711		3074	95.655			
Vector	No AA	1525	113.858	27.147	3074	97.414	1.759	0.064795373	0.1028606
HER2	No AA	1525	115.172	28.461	3074	97.556	1.901	0.066793156	0.10603202
Vector	No AA+CQ	1525	116.176	29.465	3074	114.216	18.561	0.62993382	1
HER2	No AA+CQ	1525	125.767	39.056	3074	142.557	46.902	1.200891028	1.9063765

Uncropped Western blots for Figure 3A

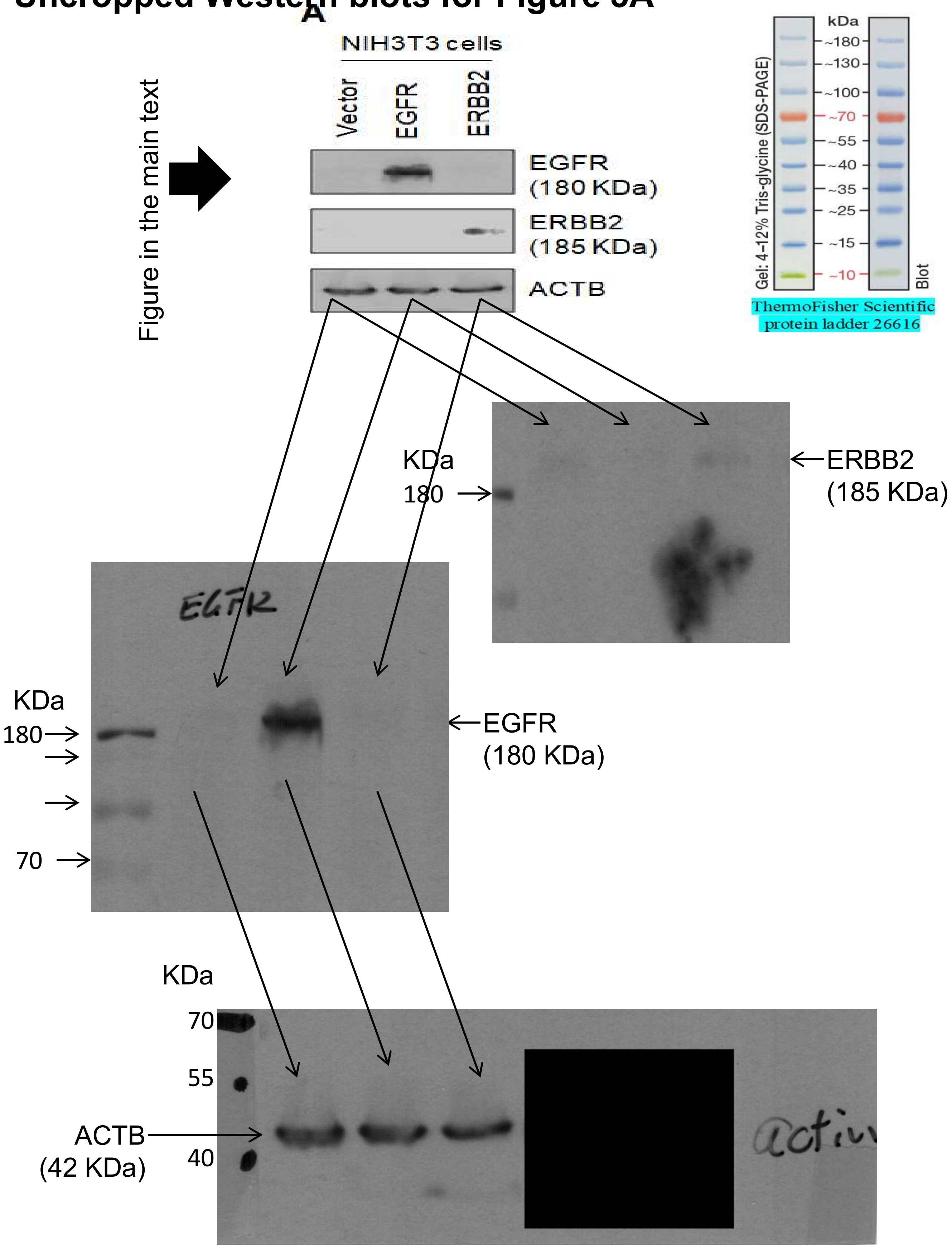

Uncropped Western blots for Figure 3B

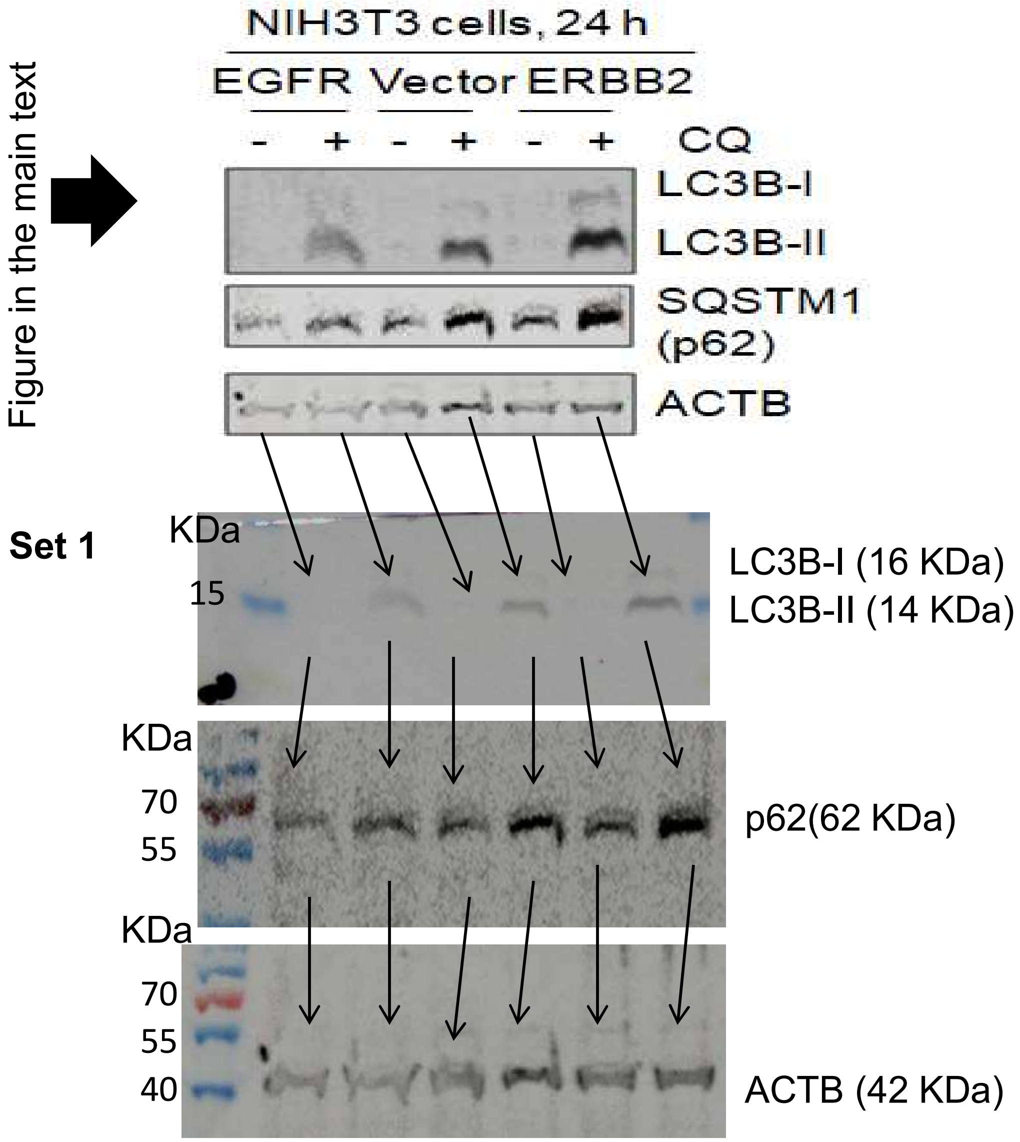

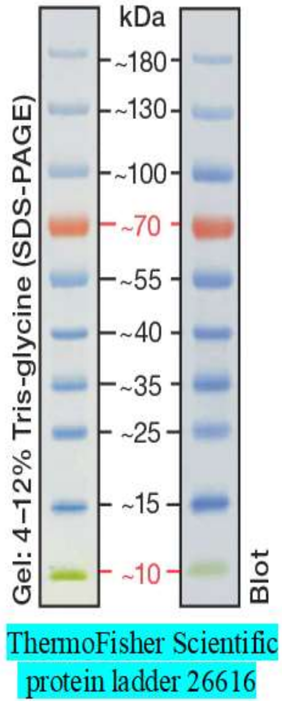

Set(1)			ACTB	ACTB		LC3II	LC3II	LC3II/ACTB	
			Intensity	Intensity		Intensity	Intensity		Relative
		Area	Mean	(-background)	Area	Mean	(-background)		
NIH3T3,24h	background	2860	109.145		1620	96.117			
EGFR	Control	2860	147.941	38.796	1620	98.626	2.509	0.064671616	0.154708091
EGFR	CQ	2860	143.487	34.342	1620	106.657	10.54	0.306912818	0.734199938
Vector	Control	2860	141.73	32.585	1620	98.37	2.253	0.069142243	0.165402772
Vector	CQ	2860	144.399	35.254	1620	110.854	14.737	0.418023487	1
EGRBB2	Control	2860	145.477	36.332	1620	98.399	2.282	0.062809644	0.150253865
EGRBB2	CQ	2860	146.025	36.88	1620	119.082	22.965	0.622695228	1.489617803

Set(1)			ACTB	ACTB		p62	p62	p62/ACTB	
			Intensity	Intensity		Intensity	Intensity		Relative
		Area	Mean	(-background)	Area	Mean	(-background)		
NIH3T3,24h	background	2860	109.145		760	109.928			
EGFR	Control	2860	147.941	38.796	760	142.845	32.917	0.848463759	0.479993282
EGFR	CQ	2860	143.487	34.342	760	151.746	41.818	1.217692621	0.688873593
Vector	Control	2860	141.73	32.585	760	149.311	39.383	1.2086236	0.683743062
Vector	CQ	2860	144.399	35.254	760	172.245	62.317	1.767657571	1
EGRBB2	Control	2860	145.477	36.332	760	156.446	46.518	1.280358912	0.724325194
EGRBB2	CQ	2860	146.025	36.88	760	195.386	85.458	2.317190889	1.310882225

Uncropped Western blots for Figure 3B

Set 2

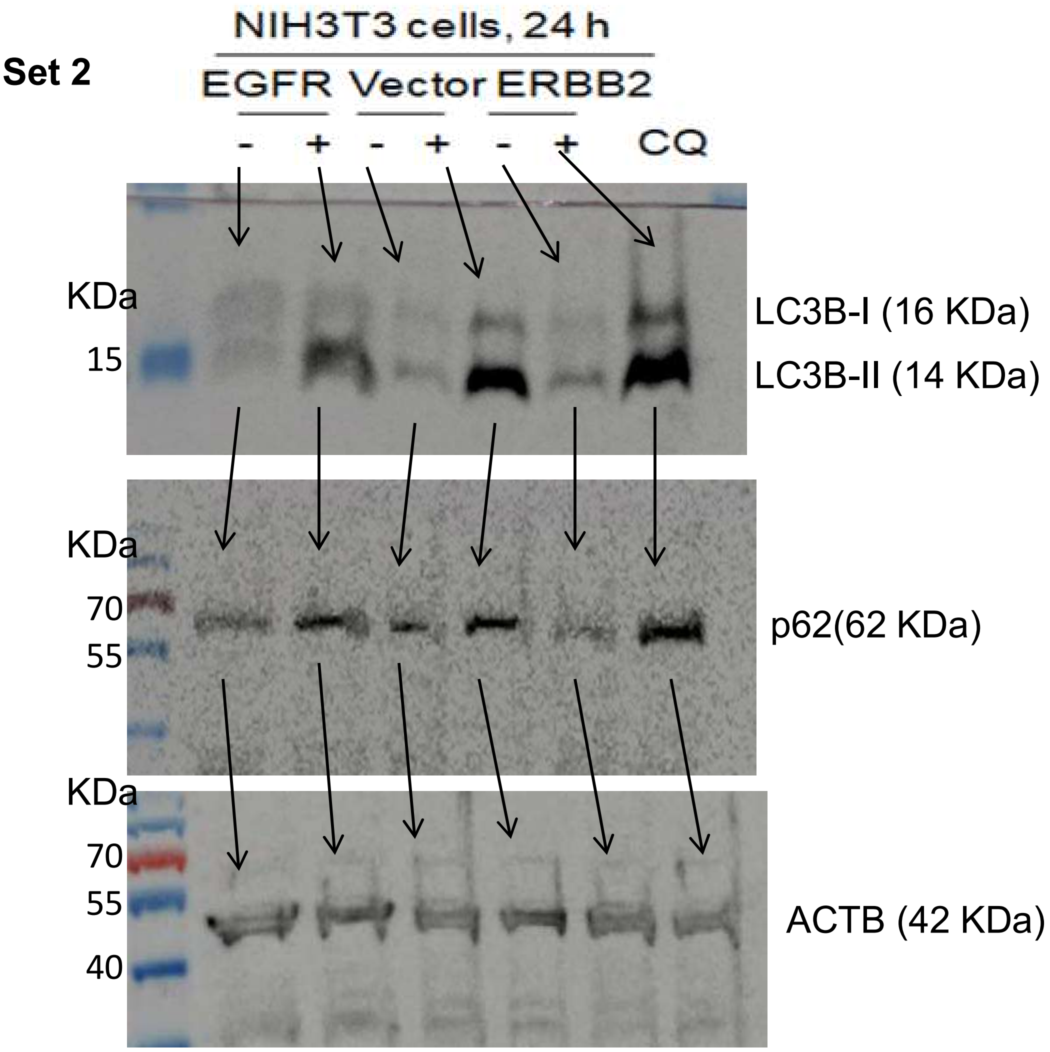

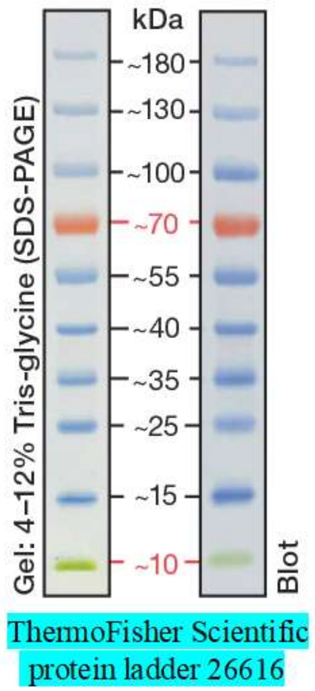

Set(2)		Area	ACTB Intensity Mean	ACTB Intensity (-background)	Area	LC3II Intensity Mean	LC3II Intensity (-background)	LC3II/ACTB	Relative
NIH3T3,24h	background	1458	102.757		2520	105.751			
EGFR	Control	1458	135.672	32.915	2520	116.46	10.709	0.325353182	0.145566081
EGFR	CQ	1458	134.75	31.993	2520	155.469	49.718	1.554027444	0.695286529
Vector	Control	1458	124.718	21.961	2520	115.688	9.937	0.452483949	0.202445584
Vector	CQ	1458	130.321	27.564	2520	167.359	61.608	2.235089247	1
EGRBB2	Control	1458	131.682	28.925	2520	120.631	14.88	0.514433881	0.230162568
EGRBB2	CQ	1458	124.757	22	2520	186.333	80.582	3.662818182	1.638779385

Set(2)		Area	ACTB Intensity Mean	ACTB Intensity (-background)	Area	p62 Intensity Mean	p62 Intensity (-background)	p62/ACTB	Relative
NIH3T3,24h	background	1458	102.757		468	110.372			
EGFR	Control	1458	135.672	32.915	468	128.385	18.013	0.547258089	0.406988505
EGFR	CQ	1458	134.75	31.993	468	142.835	32.463	1.014690714	0.754611883
Vector	Control	1458	124.718	21.961	468	130.068	19.696	0.89686262	0.666984709
Vector	CQ	1458	130.321	27.564	468	147.436	37.064	1.344652445	1
EGRBB2	Control	1458	131.682	28.925	468	122.763	12.391	0.428383751	0.318583254
EGRBB2	CQ	1458	124.757	22	468	161.374	51.002	2.318272727	1.724068354

Uncropped Western blots for Figure 3B

Set 3

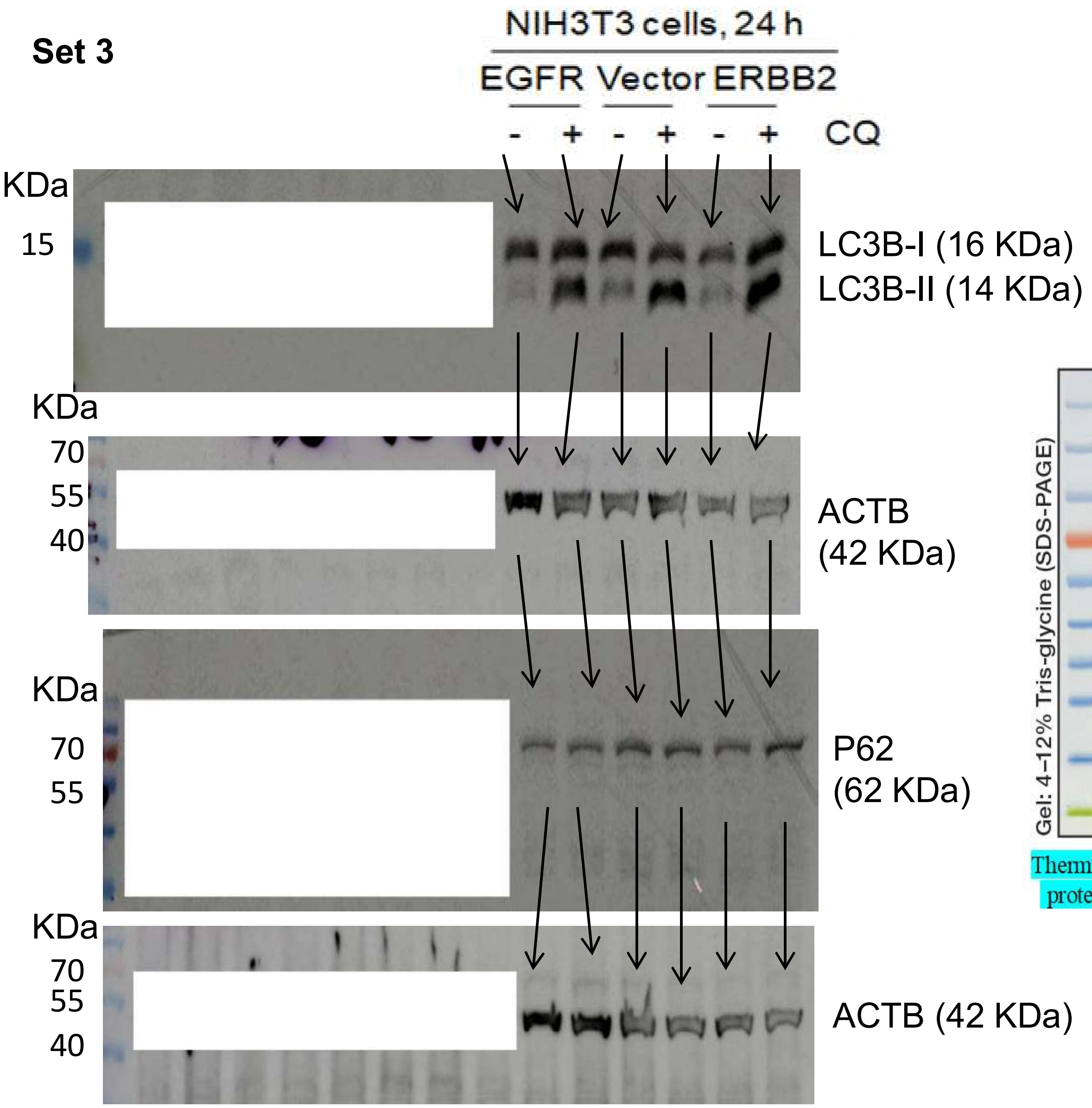

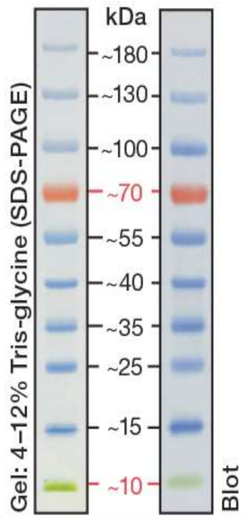

ThermoFisher Scientific protein ladder 26616

Set(3)			ACTB	ACTB		LC3II	LC3II	LC3II/ACTB	
			Intensity	Intensity		Intensity	Intensity		Relative
		Area	Mean	(-background)	Area	Mean	(-background)		
NIH3T3,24h	background	1856	100.922		2072	129			
EGFR	Control	1856	153.824	52.902	2072	136.763	7.763	0.146743034	0.096126403
EGFR	CQ	1856	136.641	35.719	2072	174.314	45.314	1.268624542	0.831033069
Vector	Control	1856	130.206	29.284	2072	148.425	19.425	0.663331512	0.434526059
Vector	CQ	1856	136.441	35.519	2072	183.222	54.222	1.526563248	1
EGRBB2	Control	1856	121.179	20.257	2072	142.826	13.826	0.682529496	0.44710201
EGRBB2	CQ	1856	124.376	23.454	2072	187.903	58.903	2.511426622	1.645150717

Set(3)			ACTB	ACTB		p62	p62	p62/ACTB	
			Intensity	Intensity		Intensity	Intensity		Relative
		Area	Mean	(-background)	Area	Mean	(-background)		
NIH3T3,24h	background	1060	97.714		500	128.12			
EGFR	Control	1060	193.054	95.34	500	159.544	31.424	0.329599329	0.349879286
EGFR	CQ	1060	171.691	73.977	500	165.338	37.218	0.503102316	0.534057759
Vector	Control	1060	157.862	60.148	500	174.29	46.17	0.76760657	0.814836728
Vector	CQ	1060	148.419	50.705	500	175.886	47.766	0.942037274	1
EGRBB2	Control	1060	146.642	48.928	500	168.15	40.03	0.818140942	0.868480435
EGRBB2	CQ	1060	133.196	35.482	500	183.328	55.208	1.555943859	1.651679717

Uncropped Western blots for Figure 3B

Figure in the main text

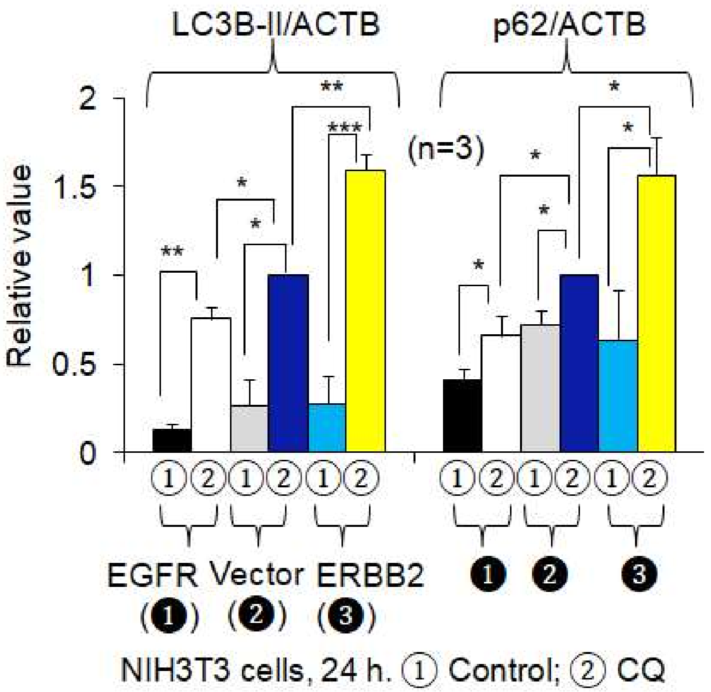

NIH3T3,24h	LC3II	Set(1)	Set(2)	Set(3)	mean	SD	p (vs vector)	p (vs control)
EGFR	Control	0.154708091	0.145566081	0.096126403	0.132133525	0.031516325		
EGFR	CQ	0.734199938	0.695286529	0.831033069	0.753506512	0.069902351	0.025775264	0.001153874
Vector	Control	0.165402772	0.202445584	0.434526059	0.267458138	0.145865726		
Vector	CQ	1	1	1	1	0		0.012960242
EGRBB2	Control	0.150253865	0.230162568	0.44710201	0.275839481	0.153604982		
EGRBB2	CQ	1.489617803	1.638779385	1.645150717	1.591182635	0.088015395	0.007307551	0.000746645
NIH3T3,24h	p62	Set(1)	Set(2)	Set(3)				
EGFR	Control	0.479993	0.406989	0.349879	0.412287	0.065218593		
EGFR	CQ	0.688874	0.754612	0.534058	0.659181333	0.113235408	0.034881795	0.04262859
Vector	Control	0.683743	0.666985	0.814837	0.721855	0.080959539		
Vector	CQ	1	1	1	1	0		0.027097863
EGRBB2	Control	0.724325	0.318583	0.86848	0.637129333	0.285129779		
EGRBB2	CQ	1.310882	1.724068	1.65168	1.56221	0.220645254	0.047698308	0.012959525

Uncropped Western blots for Figure 3C

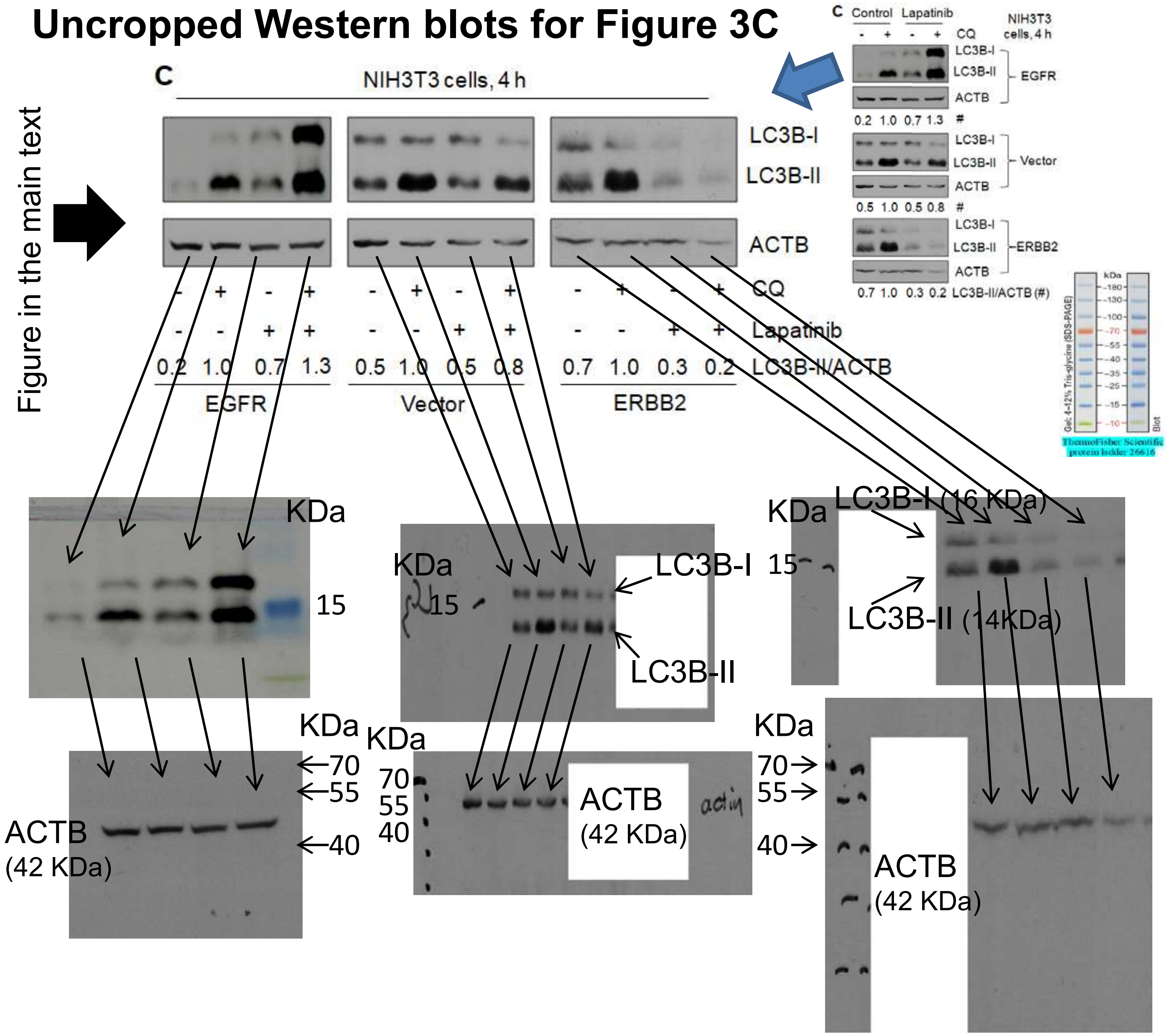

Lap=Lapatinib		ACTB Intensity	ACTB Intensity		LC3II Intensity	LC3II Intensity	LC3II/ACTB	
4 h								Relative
NIH3T3 EGFR	Area	Mean	(-background)	Area	Mean	(-background)		
background	8052	127.293		2268	109.369			
Control	8052	161.71	34.417	2268	122.23	12.861	0.373681611	0.18008777
CQ	8052	153.214	25.921	2268	163.155	53.786	2.074997107	1
1uM Lap	8052	154.838	27.545	2268	146.922	37.553	1.363332728	0.65702874
Lap+CQ	8052	159.379	32.086	2268	193.136	83.767	2.610702487	1.25817163
		ACTB Intensity	ACTB Intensity		LC3II Intensity	LC3II Intensity	LC3II/ACTB	
4 h								Relative
NIH3T3 vector alone	Area	Mean	(-background)	Area	Mean	(-background)		
background	11748	124.678		23920	128.554			
Control	11748	173.3	48.622	23920	156.957	28.403	0.584159434	0.49451153
CQ	11748	162.414	37.736	23920	173.131	44.577	1.181285775	1
1uM Lap	11748	158.694	34.016	23920	149.633	21.079	0.619678975	0.52458007
Lap+CQ	11748	159.358	34.68	23920	159.494	30.94	0.892156863	0.7552422
		ACTB Intensity	ACTB Intensity		LC3II Intensity	LC3II Intensity	LC3II/ACTB	
4 h								Relative
NIH3T3 ERBB2	Area	Mean	(-background)	Area	Mean	(-background)		
background	3003	87.403		19224	127.411			
Control	3003	143.478	56.075	19224	152.046	24.635	0.439322336	0.74310747
CQ	3003	152.148	64.745	19224	165.688	38.277	0.591196231	1
1uM Lap	3003	145.636	58.233	19224	136.097	8.686	0.149159411	0.25230102
Lap+CQ	3003	127.398	39.995	19224	132.077	4.666	0.116664583	0.19733648

Uncropped Western blots for Figure 4B (ULK1)

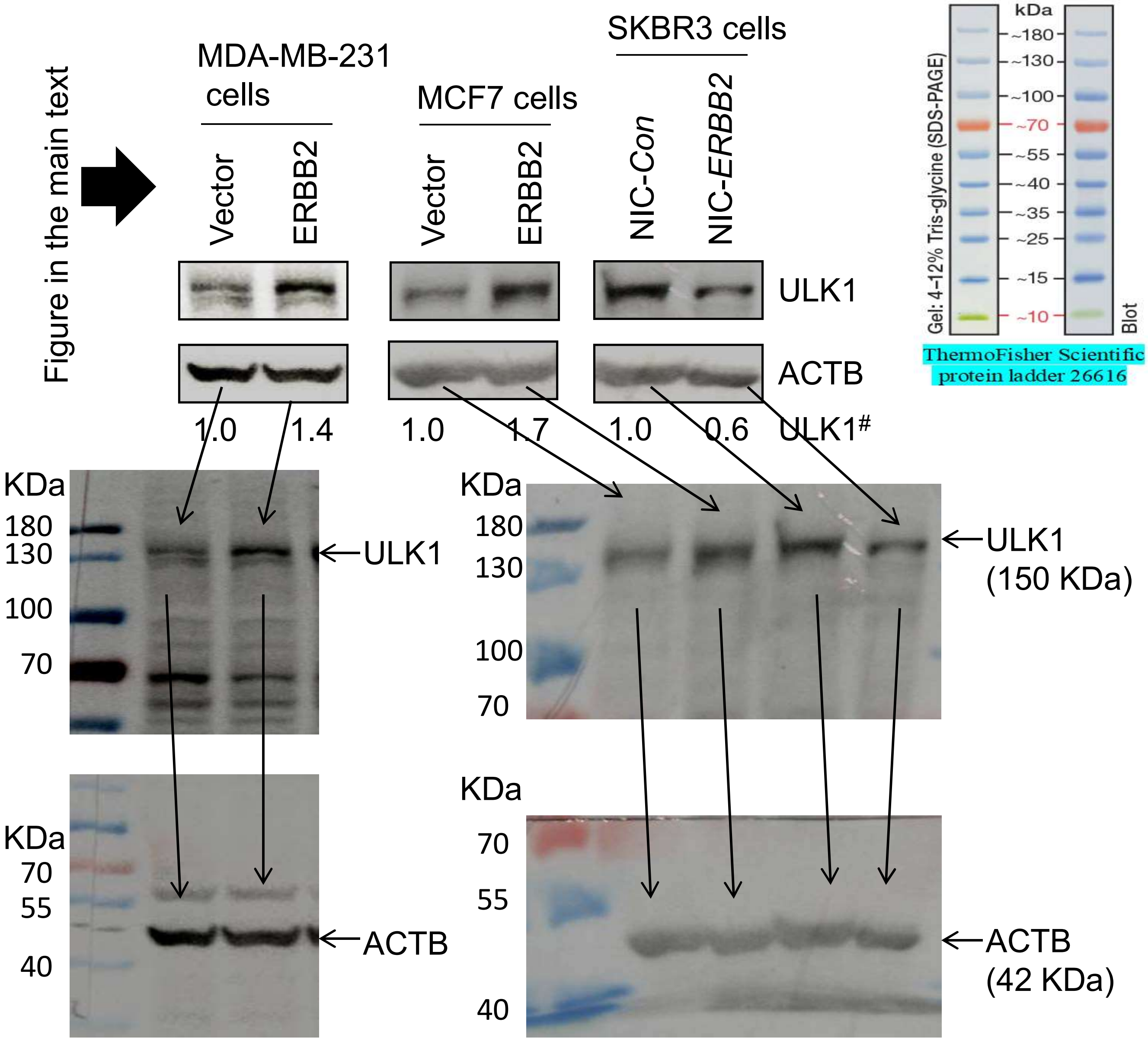

		ACTB	ACTB		ULK1	ULK1	ULK1/ACTB	
		Intensity	Intensity		Intensity	Intensity		Relative
MDA-MB-231	Area	Mean	(-background)	Area	Mean	(-background)		
background	2394	111.627		1272	117.599			
Vector	2394	171.049	59.422	1272	177.825	60.226	1.013530342	1
ERBB2	2394	169.591	57.964	1272	196.907	79.308	1.368228556	1.349963
		ACTB	ACTB		ULK1	ULK1	ULK1/ACTB	
		Intensity	Intensity		Intensity	Intensity		Relative
MCF7 cells	Area	Mean	(-background)	Area	Mean	(-background)		
background	1911	91.559		3348	96.592			
Vector	1911	129.607	38.048	3348	129.865	33.273	0.874500631	1
ERBB2	1911	124.538	32.979	3348	146.137	49.545	1.502319658	1.717917
		ACTB	ACTB		ULK1	ULK1	ULK1/ACTB	
		Intensity	Intensity		Intensity	Intensity		Relative
SKBR3	Area	Mean	(-background)	Area	Mean	(-background)		
background	1911	91.559		3348	96.592			
NIC-Con	1911	131.246	39.687	3348	150.851	54.259	1.36717313	1
NIC-ERBB2	1911	128.259	36.7	3348	128.656	32.064	0.873678474	0.63904

Uncropped Western blots for Figure 4B (FIP200)

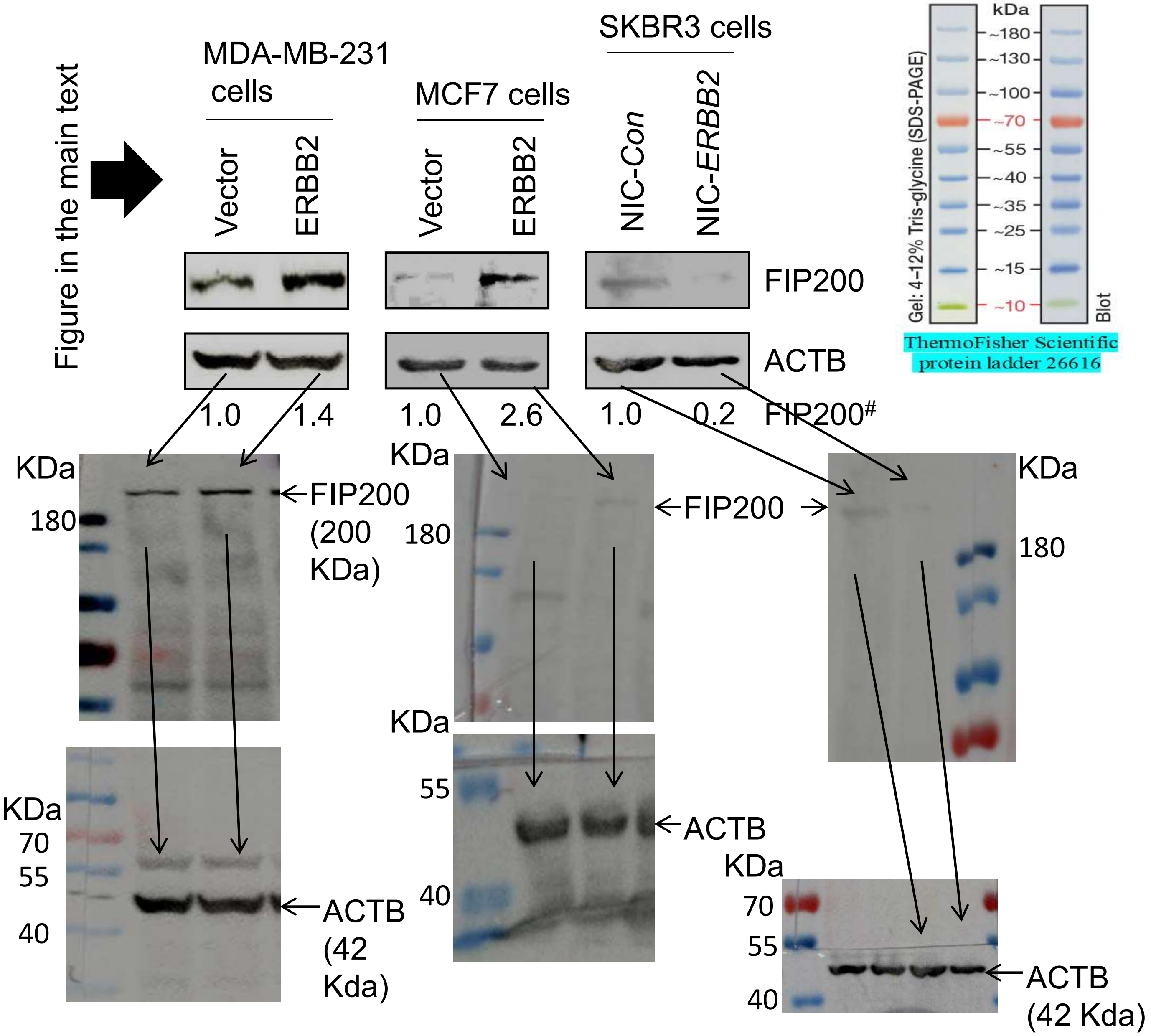

		ACTB Intensity	ACTB Intensity		FIP200 Intensity	FIP200 Intensity	FIP200/ACTB	Relative
MDA-MB-231	Area	Mean	(-background)	Area	Mean	(-background)		
background	2394	111.627		950	114.742			
Vector	2394	171.049	59.422	950	149.474	34.732	0.584497324	1
ERBB2	2394	169.591	57.964	950	161.801	47.059	0.811865986	1.3889986
		ACTB Intensity	ACTB Intensity		FIP200 Intensity	FIP200 Intensity	FIP200/ACTB	Relative
MCF7 cells	Area	Mean	(-background)	Area	Mean	(-background)		
background	2479	132.262		1464	112.839			
Vector	2479	169.804	37.542	1464	114.874	2.035	0.054205956	1
ERBB2	2479	156.468	24.206	1464	116.283	3.444	0.142278774	2.6247812
		ACTB Intensity	ACTB Intensity		FIP200 Intensity	FIP200 Intensity	FIP200/ACTB	Relative
SKBR3	Area	Mean	(-background)	Area	Mean	(-background)		
background	3965	93.387		1701	111.576			
NIC-Con	3965	138.026	44.639	1701	120.092	8.516	0.190774883	1
NIC-ERBB2	3965	129.134	35.747	1701	113.243	1.667	0.046633284	0.2444414

Uncropped Western blots for Figure 4B (BECN1)

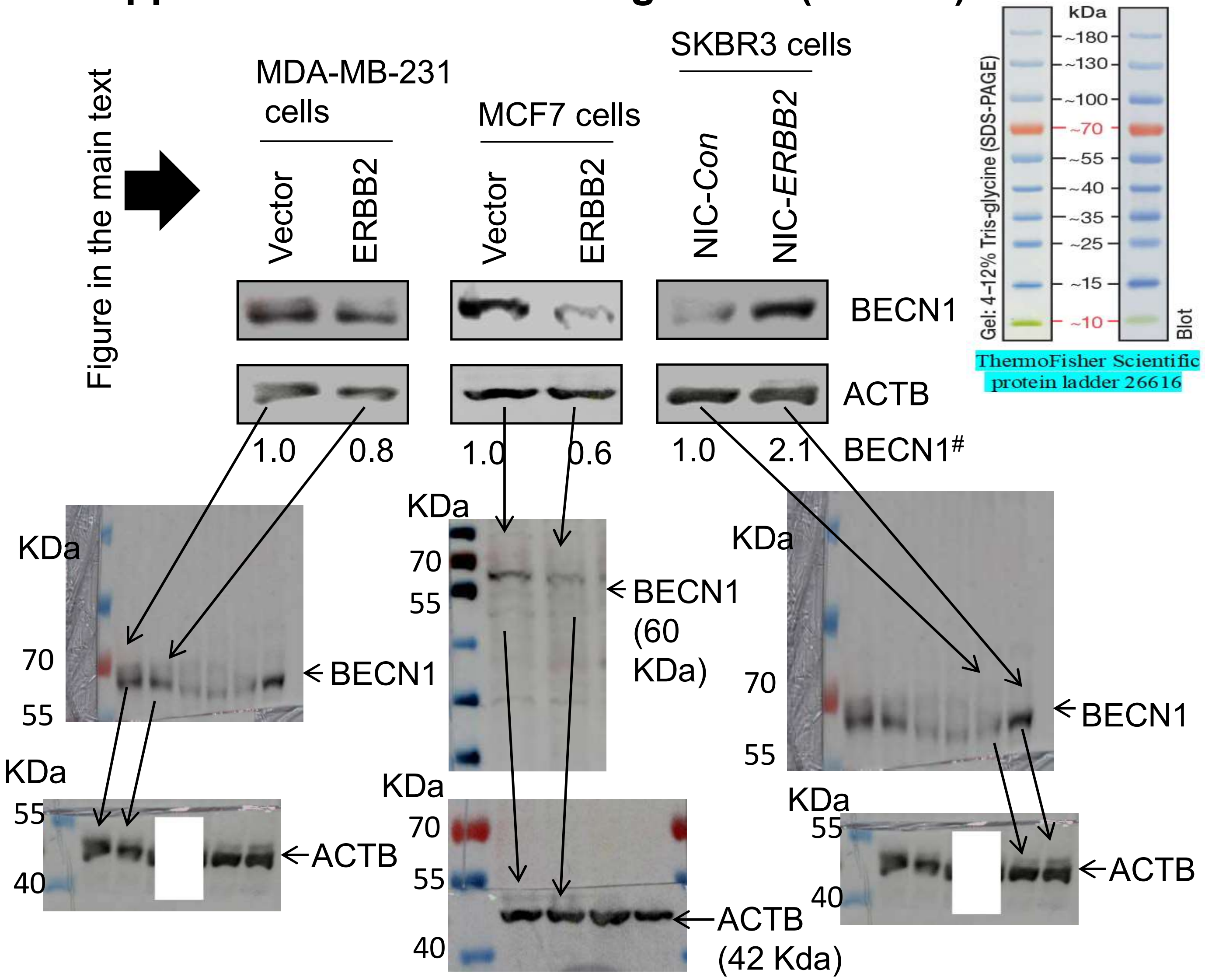

		ACTB	ACTB		BECN1	BECN1	BECN1/ACTB	
		Intensity	Intensity		Intensity	Intensity		Relative
MDA-MB-231	Area	Mean	(-background)	Area	Mean	(-background)		
background	2976	95.377		3550	97.855			
Vector	2976	162.46	67.083	3550	147.603	49.748	0.741588778	1
ERBB2	2976	152.14	56.763	3550	130.889	33.034	0.581963603	0.784752
		ACTB	ACTB		BECN1	BECN1	BECN1/ACTB	
		Intensity	Intensity		Intensity	Intensity		Relative
MCF7 cells	Area	Mean	(-background)	Area	Mean	(-background)		
background	3965	93.387		1392	85.08			
Vector	3965	132.914	39.527	1392	136.999	51.919	1.313507223	1
ERBB2	3965	130.714	37.327	1392	115.477	30.397	0.814343505	0.619976
		ACTB	ACTB		BECN1	BECN1	BECN1/ACTB	
		Intensity	Intensity		Intensity	Intensity		Relative
SKBR3	Area	Mean	(-background)	Area	Mean	(-background)		
background	2976	95.377		3550	97.855			
NIC-Con	2976	167.621	72.244	3550	114.8	16.945	0.23455235	1
NIC-ERBB2	2976	167.952	72.575	3550	133.688	35.833	0.493737513	2.105021

Uncropped Western blots for Figure 4B (ATG7)

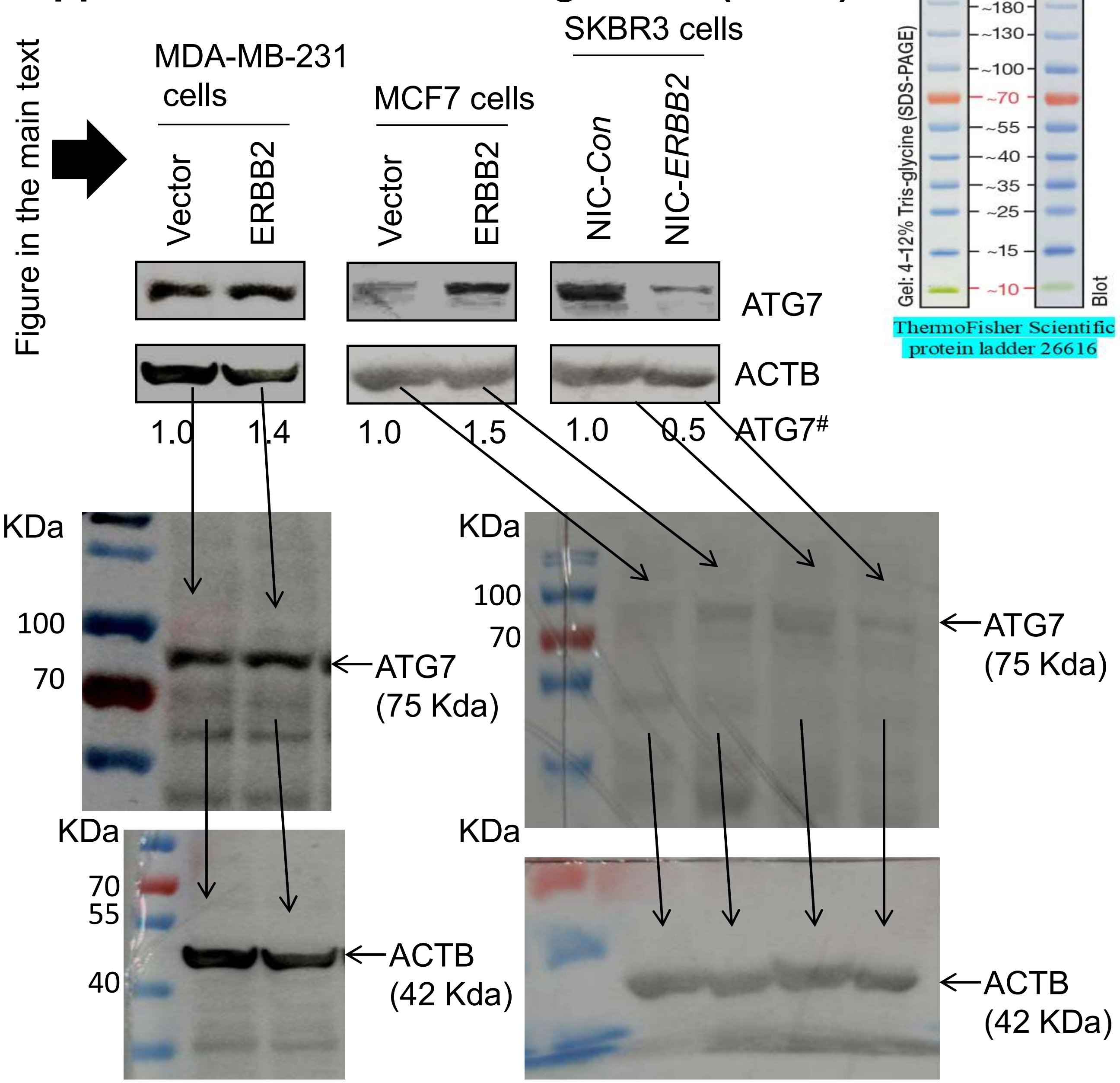

		ACTB Intensity	ACTB Intensity		ATG7 Intensity	ATG7 Intensity	ATG7/ACTB	Relative
MDA-MB-231	Area	Mean	(-background)	Area	Mean	(-background)		
background	2640	106.991		2520	115.757			
Vector	2640	194.656	87.665	2520	174.17	58.413	0.666320652	1
ERBB2	2640	170.234	63.243	2520	173.677	57.92	0.915832582	1.3744622
		ACTB Intensity	ACTB Intensity		ATG7 Intensity	ATG7 Intensity	ATG7/ACTB	Relative
MCF7 cells	Area	Mean	(-background)	Area	Mean	(-background)		
background	1911	91.559		1568	120.983			
Vector	1911	129.607	38.048	1568	131.138	10.155	0.266899706	1
ERBB2	1911	124.538	32.979	1568	133.708	12.725	0.385851603	1.4456801
		ACTB Intensity	ACTB Intensity		ATG7 Intensity	ATG7 Intensity	ATG7/ACTB	Relative
SKBR3	Area	Mean	(-background)	Area	Mean	(-background)		
background	1911	91.559		1568	120.983			
NIC-Con	1911	131.246	39.687	1568	138.55	17.567	0.442638647	1
NIC-ERBB2	1911	128.259	36.7	1568	129.593	8.61	0.234604905	0.5300145

Uncropped Western blots for Figure 4B (ATG5)

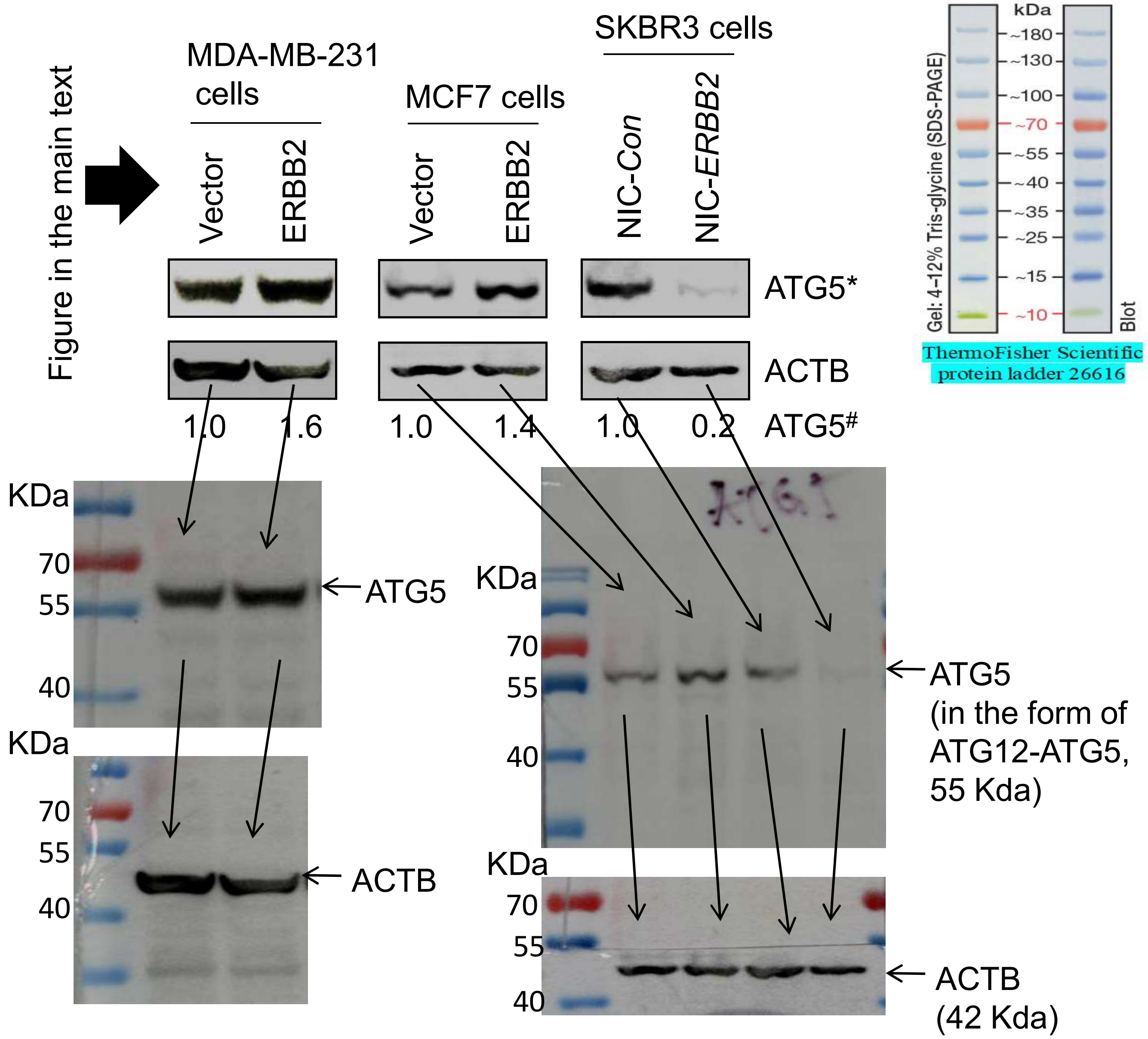

		ACTB	ACTB		ATG5	ATG5	ATG5/ACTB	
		Intensity	Intensity		Intensity	Intensity		Relative
MDA-MB-231	Area	Mean	(-background)	Area	Mean	(-background)		
background	2640	106.991		2457	115.218			
Vector	2640	194.656	87.665	2457	162.941	47.723	0.544379171	1
ERBB2	2640	170.234	63.243	2457	171.81	56.592	0.894834211	1.64377
		ACTB	ACTB		ATG5	ATG5	ATG5/ACTB	
		Intensity	Intensity		Intensity	Intensity		Relative
MCF7 cells	Area	Mean		Area	Mean	(-background)		
background	3965	93.387		1767	110.865			
Vector	3965	132.914	39.527	1767	134.502	23.637	0.597996306	1
ERBB2	3965	130.714	37.327	1767	142.81	31.945	0.855814826	1.431137
		ACTB	ACTB		ATG5	ATG5	ATG5/ACTB	
		Intensity	Intensity		Intensity	Intensity		Relative
SKBR3	Area	Mean	(-background)	Area	Mean	(-background)		
background	3965	93.387		1767	110.865			
NIC-Con	3965	138.026	44.639	1767	131.495	20.63	0.46215193	1
NIC-ERBB2	3965	129.134	35.747	1767	114.074	3.209	0.089769771	0.194243

Uncropped Western blots for Figure 4B (ATG12)

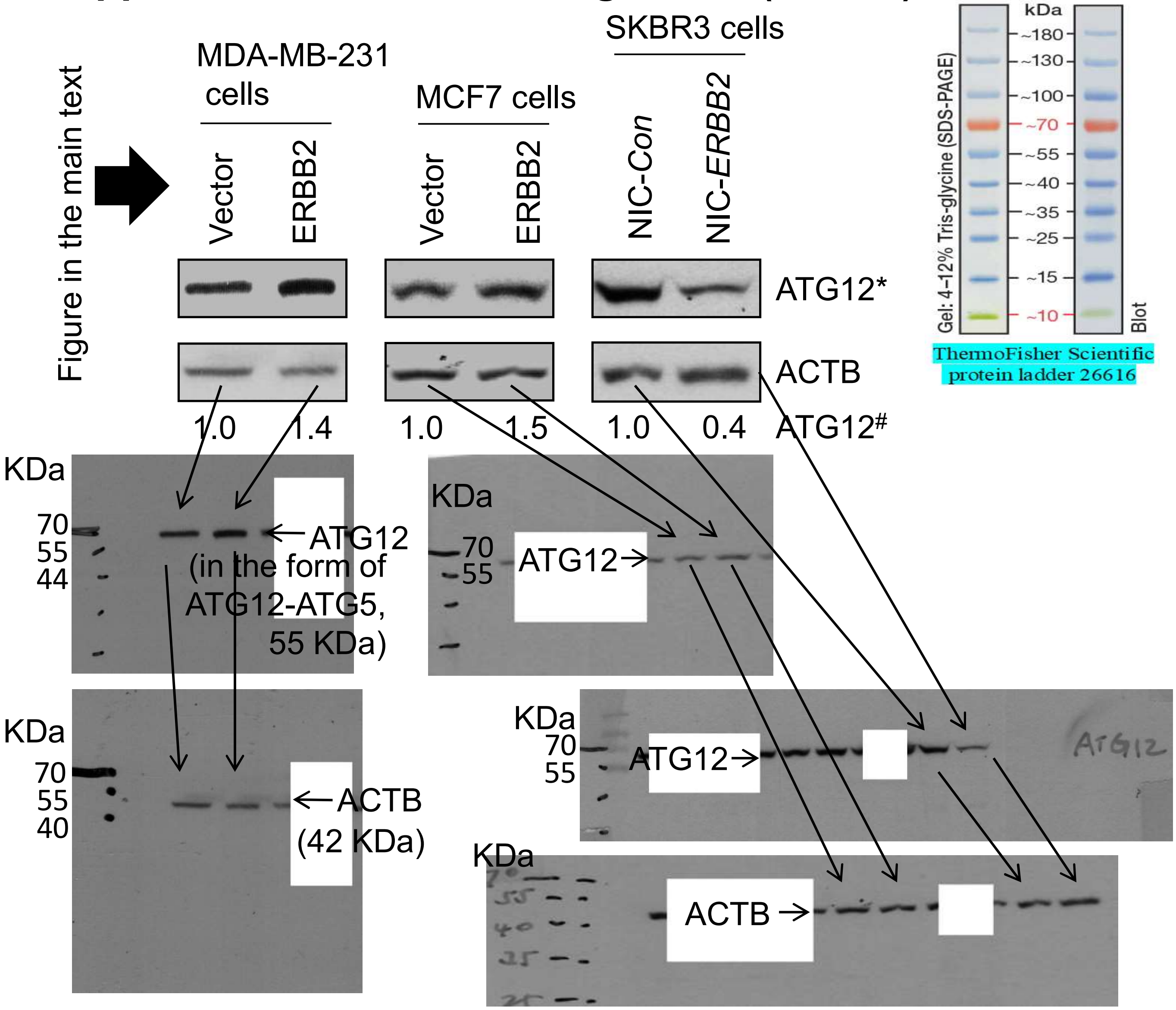

		ACTB Intensity Mean	ACTB Intensity (-background)		ATG12 Intensity Mean	ATG12 Intensity (-background)	ATG12/ACTB	Relative
MDA-MB-231	Area			Area				
background	7832	127.008		9360	135.352			
Vector	7832	162.611	35.603	9360	166.669	31.317	0.879616886	1
ERBB2	7832	157.16	30.152	9360	172.219	36.867	1.222704962	1.3900426
		ACTB Intensity	ACTB Intensity		ATG12 Intensity	ATG12 Intensity	ATG12/ACTB	Relative
MCF7 cells	Area	Mean	(-background)	Area	Mean	(-background)		
background	7680	112.52		7424	114.856			
Vector	7680	149.136	36.616	7424	127.239	12.383	0.338185493	1
ERBB2	7680	143.973	31.453	7424	131.145	16.289	0.517883827	1.5313603
		ACTB Intensity	ACTB Intensity		ATG12 Intensity	ATG12 Intensity	ATG12/ACTB	Relative
SKBR3	Area	Mean	(-background)	Area	Mean	(-background)		
background	7680	112.52		7424	114.856			
NIC-Con	7680	142.179	29.659	7424	127.73	12.874	0.434067231	1
NIC-ERBB2	7680	151.204	38.684	7424	121.676	6.82	0.176300279	0.4061589

Uncropped Western blots for Figure 6A

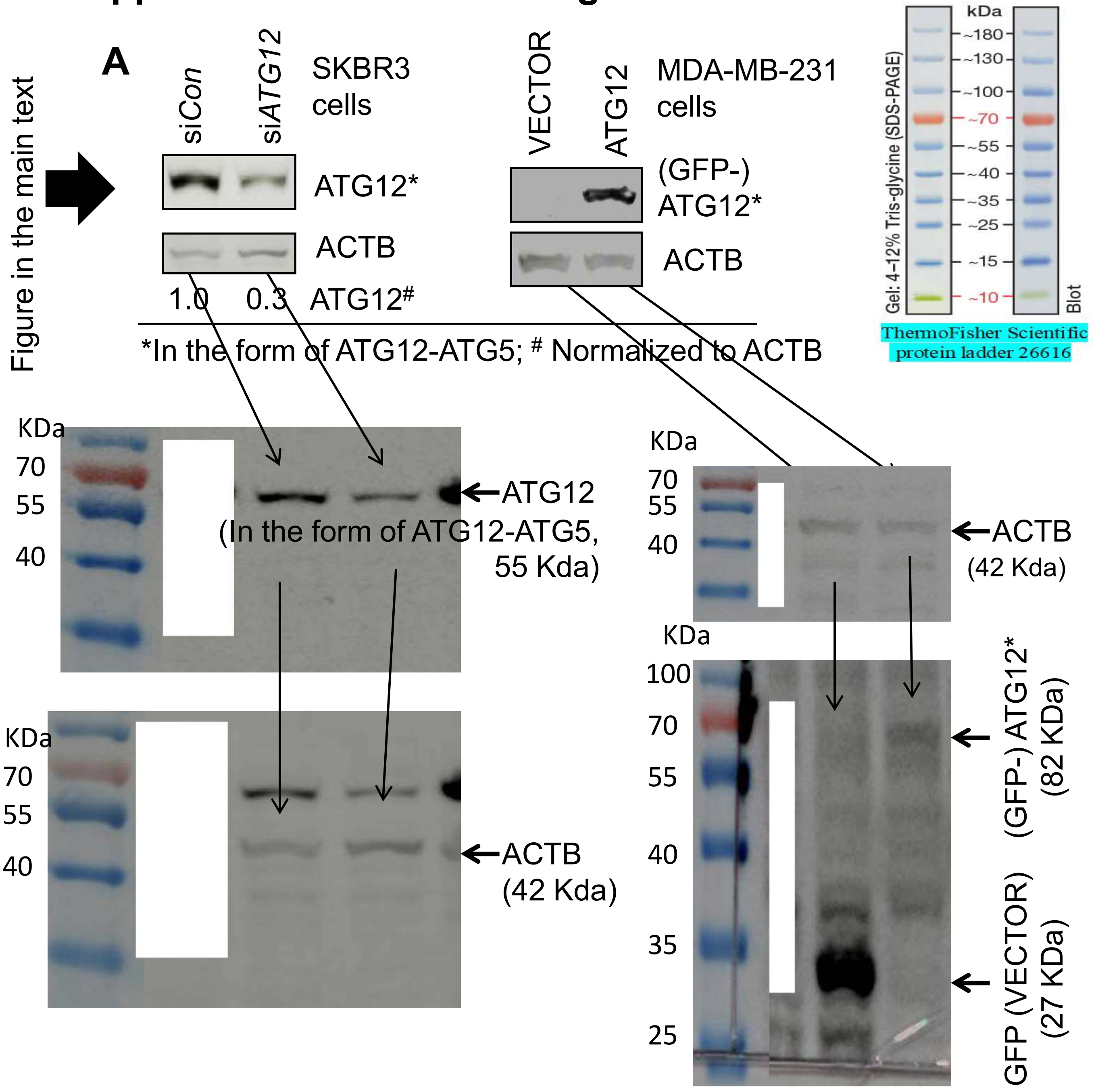

SKBR3	Area	ACTB Intensity Mean	ACTB Intensity (-background)	Area	ATG12 Intensity Mean	ATG12 Intensity (-background)	ATG12/ACTB	Relative
background	2160	114.288		1736	115.869			
siCon	2160	120.488	6.2	1736	136.767	20.898	3.370645161	1
siATG12	2160	122.225	7.937	1736	124.903	9.034	1.138213431	0.337684

Uncropped Western blots for Figure 6B

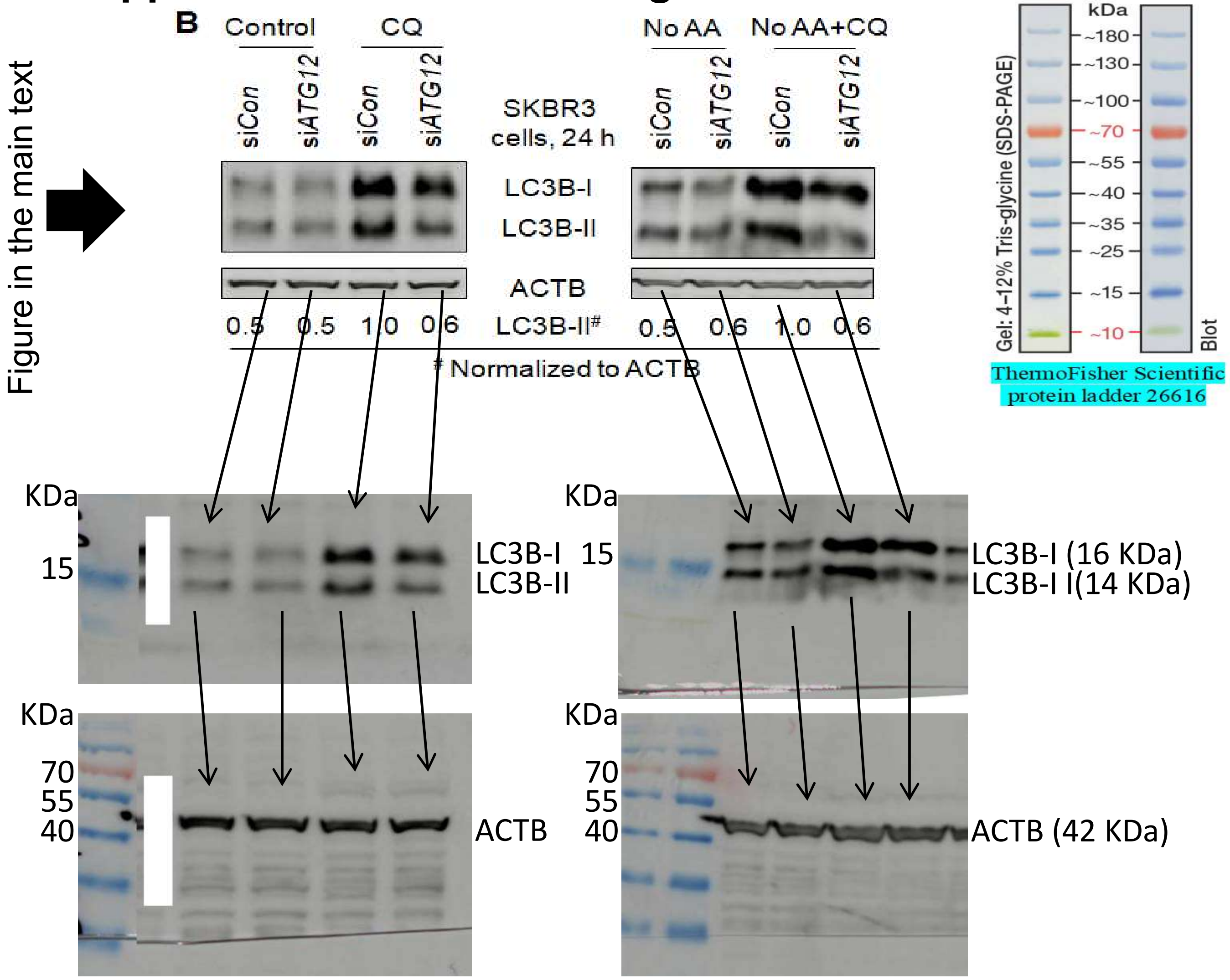

24 h SKBR3	Area	ACTB Intensity Mean	ACTB Intensity (-background)	Area	LC3II Intensity Mean	LC3II Intensity (-background)	LC3II/ACTB	Relative
background	1612	92.79		2146	95.057			
siCon	1612	161.129	68.339	2146	112.813	17.756	0.259822356	0.457365
siATG12	1612	162.426	69.636	2146	113.713	18.656	0.267907404	0.471597
siCon+CQ	1612	157.613	64.823	2146	131.882	36.825	0.568085402	1
siATG12+CQ	1612	157.621	64.831	2146	115.993	20.936	0.322931931	0.568457
24 h SKBR3		ACTB Intensity Mean	ACTB Intensity (-background)		LC3II Intensity Mean	LC3II Intensity (-background)	LC3II/ACTB	Relative
background	2048	93.457		2432	94.582			
siCon+No AA	2048	148.904	55.447	2432	131.086	36.504	0.658358432	0.453636
siATG12+No AA	2048	148.595	55.138	2432	143.246	48.664	0.882585513	0.608137
siCon+No AA+CQ	2048	149.917	56.46	2432	176.522	81.94	1.451292951	1
siATG12+No AA+CQ	2048	151.101	57.644	2432	143.85	49.268	0.854694331	0.588919

Uncropped Western blots for Figure 6C

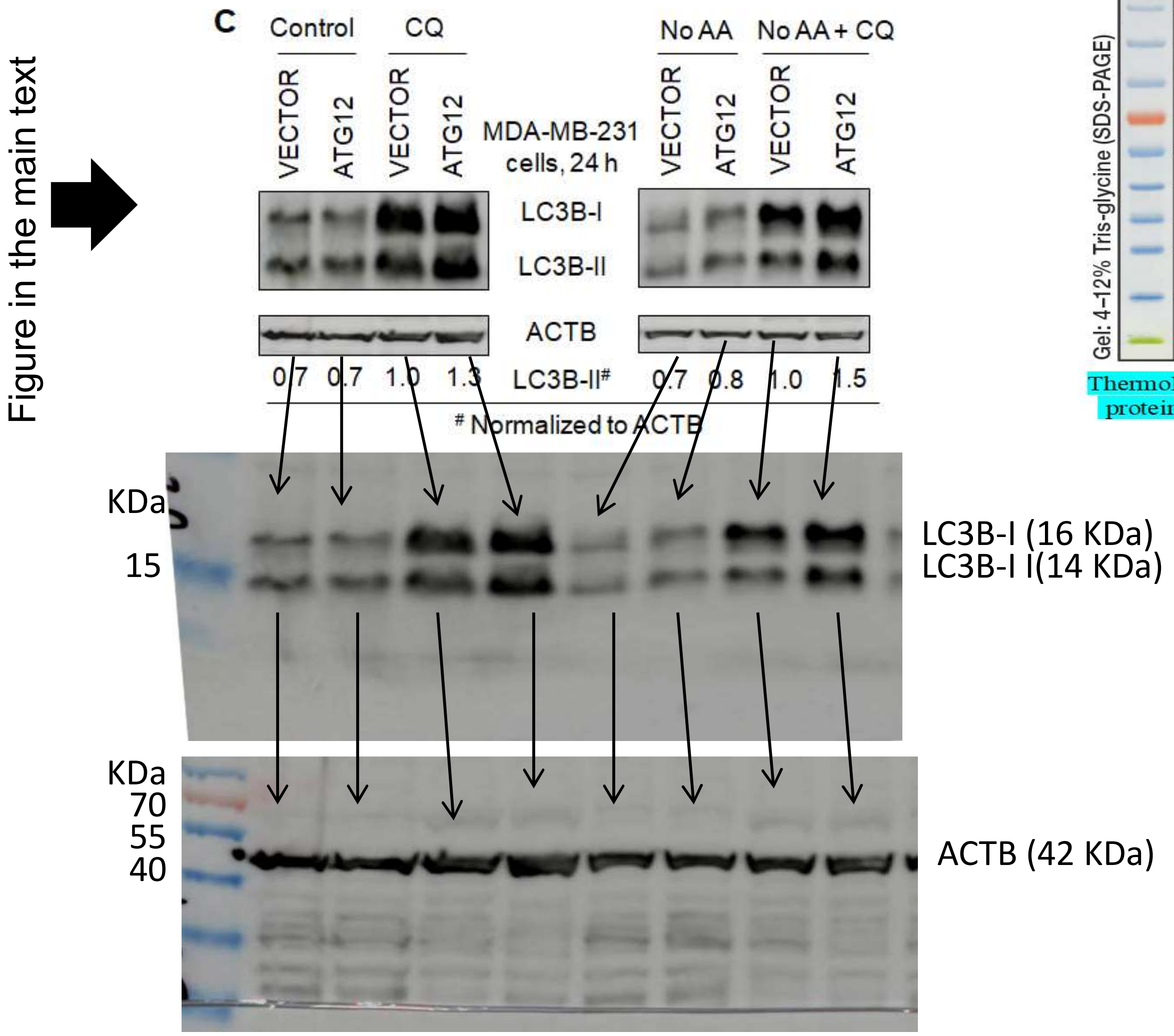

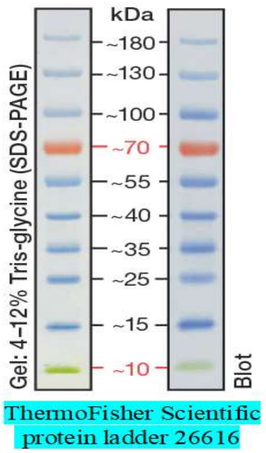

		ACTB	ACTB		LC3II	LC3II	LC3II/ACTB	
24 h		Intensity	Intensity		Intensity	Intensity		Relative
MDA-MB-231 cells	Area	Mean	(-background)	Area	Mean	(-background)		
background	1612	92.79		2146	95.057			
VECTOR	1612	187.523	94.733	2146	127.892	32.835	0.346605723	0.652937
ATG12	1612	176.122	83.332	2146	127.697	32.64	0.391686267	0.73786
VECTOR+CQ	1612	188.344	95.554	2146	145.781	50.724	0.5308412	1
ATG12+CQ	1612	191.205	98.415	2146	160.406	65.349	0.664014632	1.250872
		ACTB	ACTB		LC3II	LC3II	LC3II/ACTB	
24 h		Intensity	Intensity		Intensity	Intensity		Relative
MDA-MB-231 cells		Mean	(-background)		Mean	(-background)		
background	1612	92.79		2146	95.057			
VECTOR+No AA	1612	164.322	71.532	2146	114.664	19.607	0.274101102	0.718804
ATG12+No AA	1612	163.433	70.643	2146	115.411	20.354	0.288124797	0.75558
VECTOR+No AA+CQ	1612	166.532	73.742	2146	123.177	28.12	0.3813295	1
ATG12+No AA+CQ	1612	163.858	71.068	2146	136.208	41.151	0.579036979	1.518469

Uncropped Western blots for Figure 7A

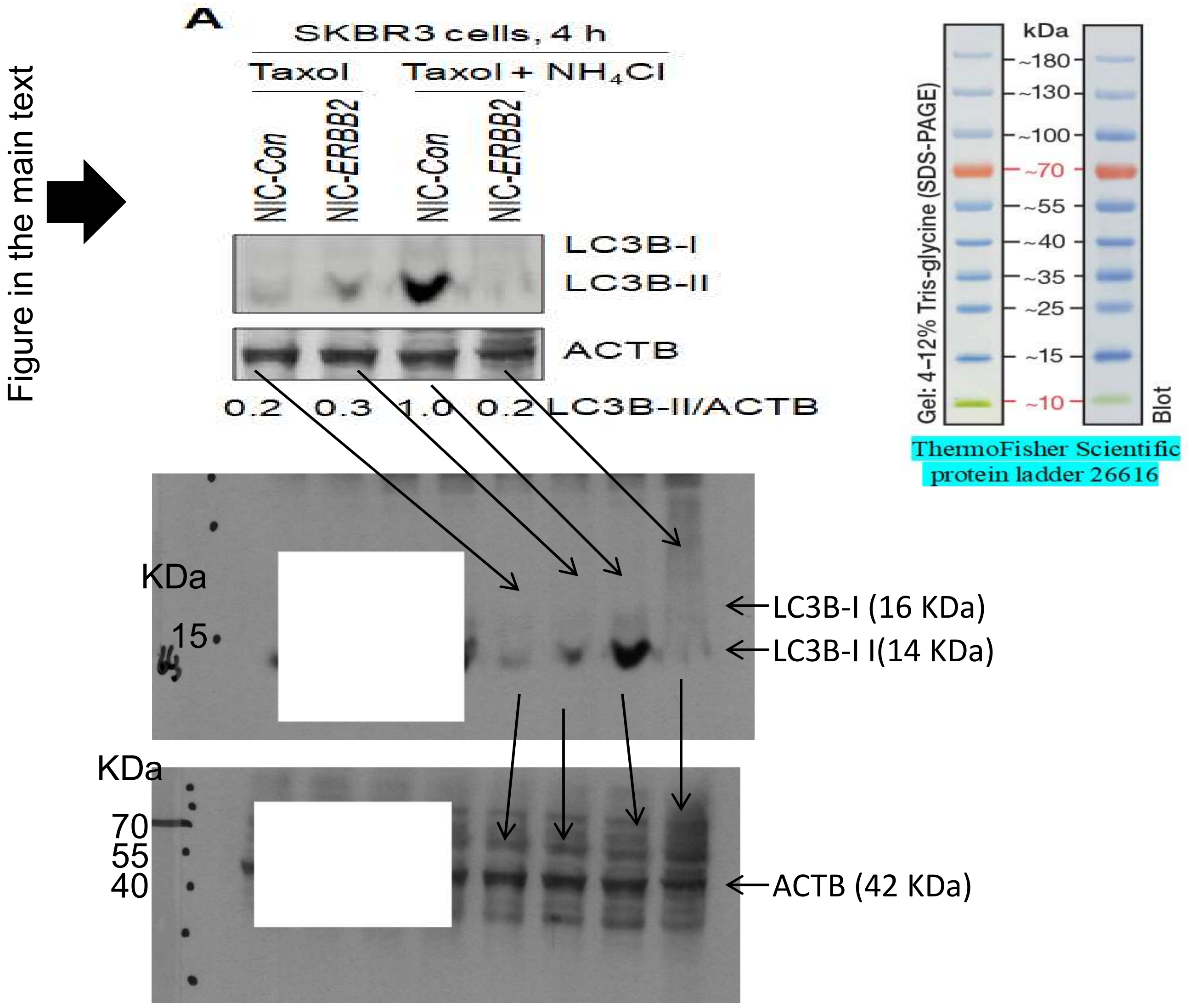

4 h	Area	ACTB Intensity Mean	ACTB Intensity (-background)	Area	LC3II Intensity Mean	LC3II Intensity (-background)	LC3II/ACTB	Relative
SKBR3								
background	11700	115.31		3444	93.862			
NIC-Con+Taxol	11700	181.019	65.709	3444	107.997	14.135	0.21511513	0.23489
NIC-ERBB2+Taxol	11700	184.265	68.955	3444	114.931	21.069	0.3055471	0.33363
NIC-Con+Taxol+NH4Cl	11700	188.185	72.875	3444	160.603	66.741	0.91582847	1
NIC-ERBB2+Taxol+NH4Cl	11700	192.219	76.909	3444	106.025	12.163	0.15814794	0.17268

Uncropped Western blots for Figure 7C

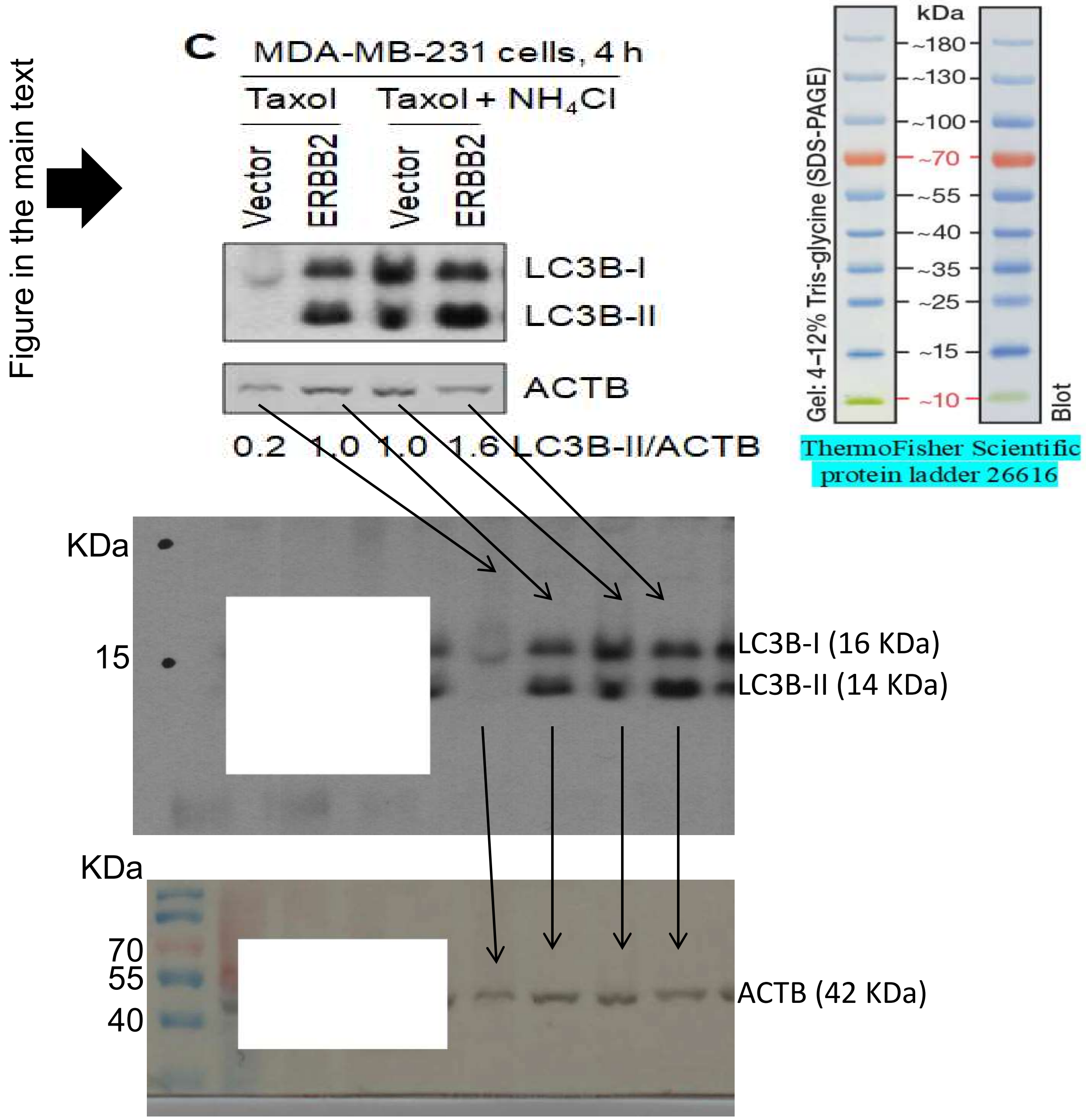

4 h	Area	ACTB Intensity Mean	ACTB Intensity (-background)	Area	LC3II Intensity Mean	LC3II Intensity (-background)	LC3II/ACTB	Relative
MDA-MB-231 cells								
background	931	120.656		14742	119.223			
Vector+Taxol	931	137.803	17.147	14742	123.677	4.454	0.25975389	0.22412
ERBB2+Taxol	931	146.69	26.034	14742	148.676	29.453	1.13132826	0.97612
Vector+Taxol+NH4Cl	931	145.617	24.961	14742	148.153	28.93	1.15900805	1
ERBB2+Taxol+NH4Cl	931	141.576	20.92	14742	158.176	38.953	1.86199809	1.60654

Uncropped Western blots for Figure 8A

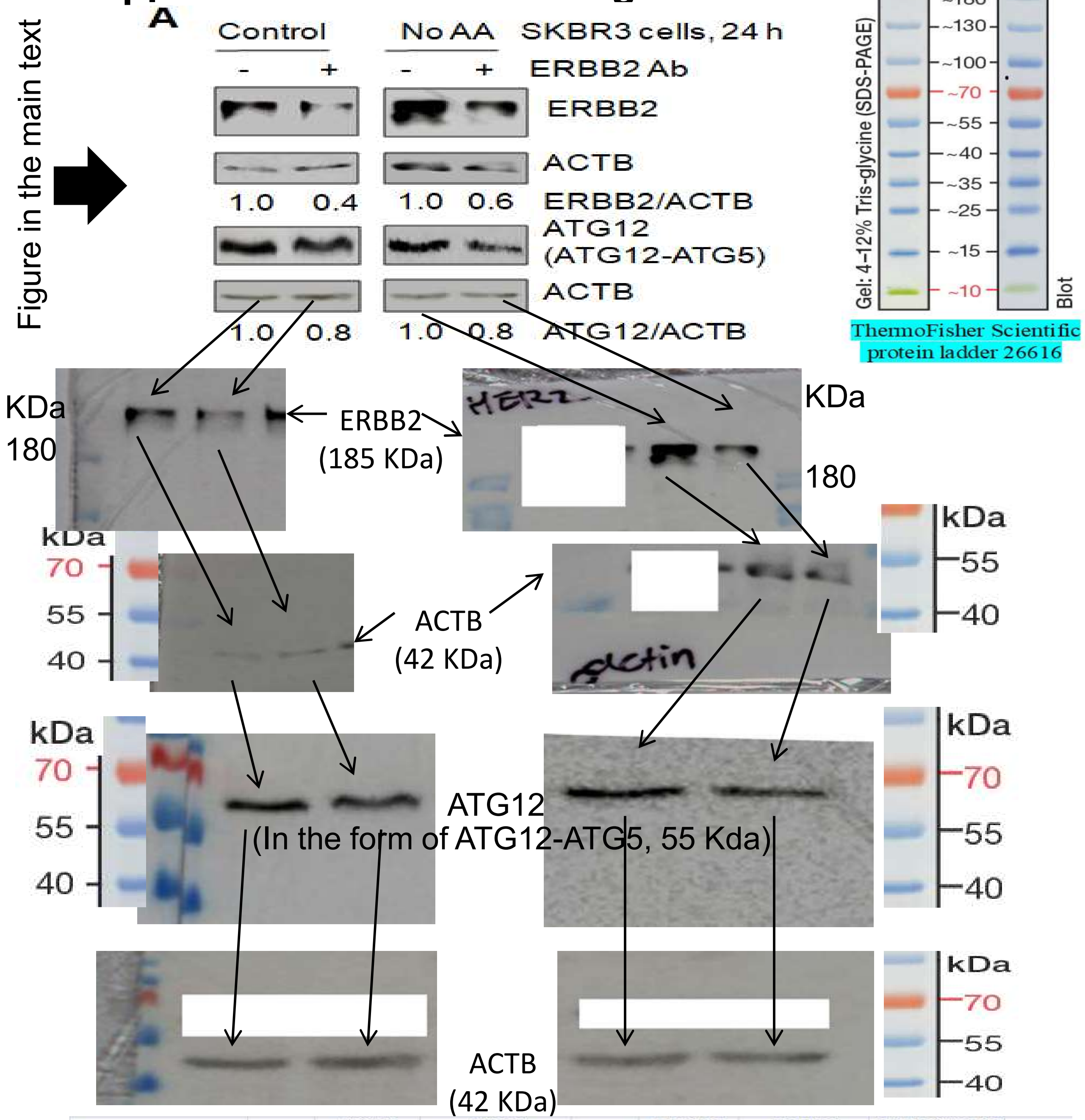

24 h	Area	ACTB Intensity	ACTB Intensity	Area	ERBB2 Intensity	ERBB2 Intensity	ERBB2/ACTB	Relative
SKBR3		Mean	(-background)		Mean	(-background)		
background	1785	132.337		3712	106.12			
Control	1785	136.336	3.999	3712	135.719	29.599	7.4016004	1
ERBB2 Ab	1785	137.818	5.481	3712	123.341	17.221	3.141944901	0.424
24 h	Area	ACTB Intensity	ACTB Intensity	Area	ERBB2 Intensity	ERBB2 Intensity	ERBB2/ACTB	Relative
SKBR3		Mean	(-background)		Mean	(-background)		
background	2706	106.572		3965	99.092			
No AA	2706	139.213	32.641	3965	169.326	70.234	2.151711038	1
No AA+ERBB2 Ab	2706	128.352	21.78	3965	127.096	28.004	1.285766758	0.598
24 h	Area	ACTB Intensity	ACTB Intensity	Area	ATG12 Intensity	ATG12 Intensity	ATG12/ACTB	Relative
SKBR3		Mean	(-background)		Mean	(-background)		
background	1368	100.659		990	93.151			
Control	1368	127.022	26.363	990	141.964	48.813	1.851572279	1
ERBB2 Ab	1368	132.212	31.553	990	138.58	45.429	1.439768009	0.778
24 h	Area	ACTB Intensity	ACTB Intensity	Area	ATG12 Intensity	ATG12 Intensity	ATG12/ACTB	Relative
SKBR3		Mean	(-background)		Mean	(-background)		
background	1368	100.659		1888	100.41			
No AA	1368	125.951	25.292	1888	132.916	32.506	1.285228531	1
No AA+ERBB2 Ab	1368	123.178	22.519	1888	122.699	22.289	0.989786403	0.77

Uncropped Western blots for Figure 8C

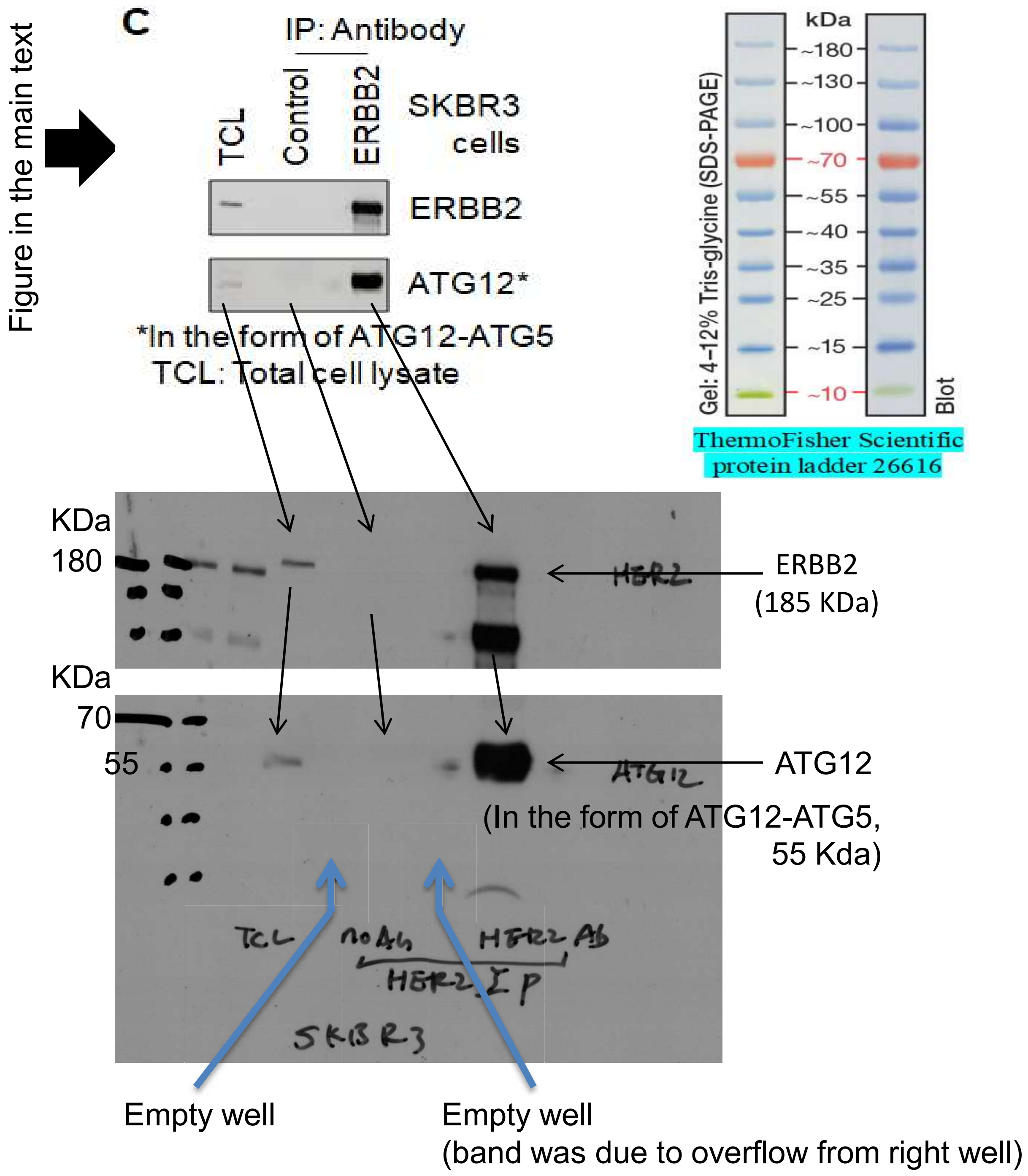

Uncropped Western blots for Figure 8D

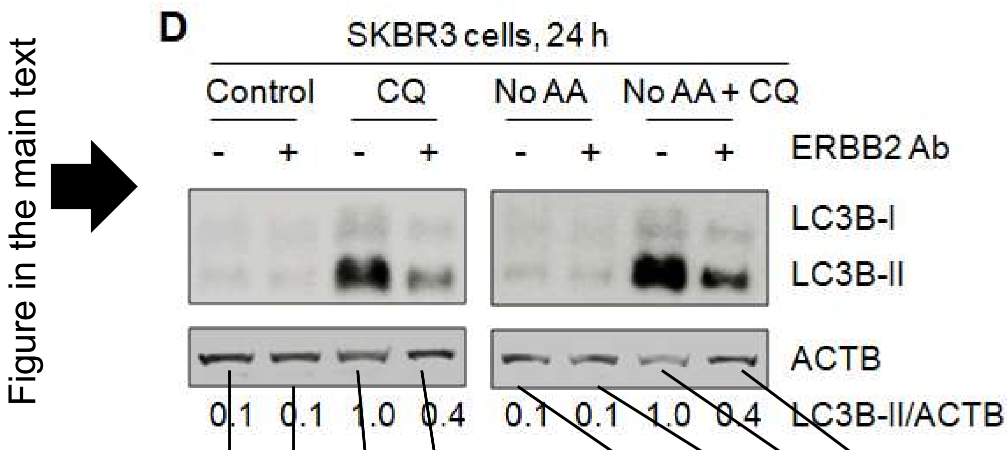

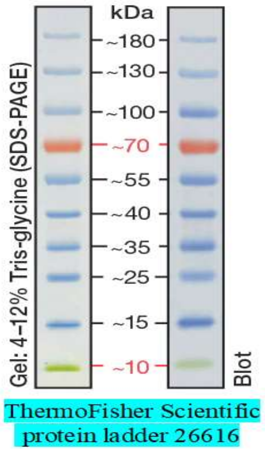

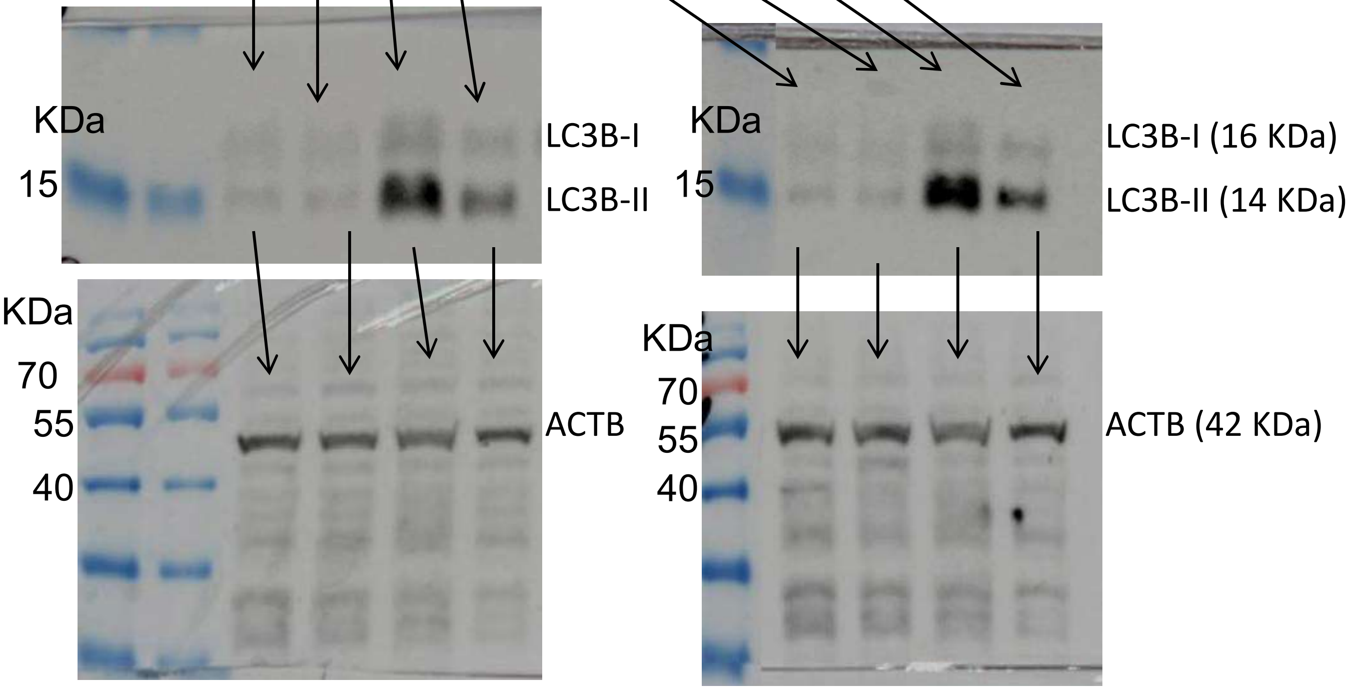

24 h	Area	ACTB Intensity Mean	ACTB Intensity (-background)	Area	LC3II Intensity Mean	LC3II Intensity (-background)	LC3II/ACTB	Relative
SKBR3								
background	1121	85.228		3328	97.43			
Control	1121	130.368	45.14	3328	102.035	4.605	0.10201595	0.079
ERBB2 Ab	1121	124.242	39.014	3328	101.565	4.135	0.105987594	0.082
CQ	1121	123.829	38.601	3328	147.306	49.876	1.292090878	1
ERBB2 Ab+CQ	1121	123.05	37.822	3328	115.15	17.72	0.468510391	0.363

24 h	Area	ACTB Intensity Mean	ACTB Intensity (-background)	Area	LC3II Intensity Mean	LC3II Intensity (-background)	LC3II/ACTB	Relative
SKBR3								
background	1525	86.711		3074	95.655			
No AA	1525	126.169	39.458	3074	101.34	5.685	0.144077247	0.063
No AA+ERBB2 Ab	1525	123.603	36.892	3074	101.697	6.042	0.163775344	0.072
No AA+CQ	1525	119.873	33.162	3074	171.271	75.616	2.280200229	1
No AA+ERBB2 Ab+CQ	1525	124.517	37.806	3074	125.936	30.281	0.80095752	0.351
